# Supplementary material for: A general one-step protocol to generate impermeable fluorescent HaloTag substrates for in situ live cell application and super-resolution imaging
Source: Nat Commun. 2026 Jan 12;17:426. doi: 10.1038/s41467-025-68134-0 (PMC12796469; doi:10.1038/s41467-025-68134-0)
Supplement: Supplementary file 1 — Supplementary Information [file 41467_2025_68134_MOESM1_ESM.pdf]

Supplementary Information for

A general one-step protocol to generate impermeable fluorescent HaloTag substrates for *in situ* live cell application and super-resolution imaging

Kilian Roßmann[1], Ulrich Pabst[1], Bianca C. Baciu[1], Siqi Sun[1], Christiane Huhn[1], Christina Holmboe Olesen[1], Maria Kowald[1], Eleni Tapp[1], Marie Bieck[1], Ramona Birke[1], Brenda C. Shields[2], Pyeonghwa Jeong[3], Jiyong Hong[3], Michael R. Tadross[2], Joshua Levitz[4], Martin Lehmann[1], Noa Lipstein[1] and Johannes Broichhagen[1,*]

[1] Leibniz-Forschungsinstitut für Molekulare Pharmakologie (FMP), 13125 Berlin, Germany.
[2] Duke University, Departments of Neurosurgery, Neurobiology and Biomedical Engineering, Durham, North Carolina 27708, USA
[3] Duke University, Department of Chemistry, Durham, North Carolina 27708, USA
[4] Weill Cornell Medicine, Department of Biochemistry, NY, USA.

* Correspondence should be addressed to:
broichhagen@fmp-berlin.de

Contents

1 Synthesis 4

1.1 *tert*-Butyl (2-(2-((6-hydroxyhexyl)oxy)ethoxy)ethyl)carbamate (BocNH-HTL^{OH}) 4

1.2 2-(6-(Dimethylamino)-3-(dimethyliminio)-3*H*-xanthen-9-yl)-4-((2-(2-((6-hydroxyhexyl)oxy)ethoxy)ethyl)carbamoyl)benzoate (TMR-HTL^{OH}) 5

1.3 2-(6-(Dimethylamino)-3-(dimethyliminio)-3H-xanthen-9-yl)-4-((2-(2-((6-(phosphonooxy)hexyl)oxy)ethoxy)ethyl)carbamoyl)benzoate (TMR-HTL^{PO4}) 5

1.4 2-(6-(Dimethylamino)-3-(dimethyliminio)-3H-xanthen-9-yl)-4-((2-(2-((6-(sulfooxy)hexyl)oxy)ethoxy)ethyl)carbamoyl)benzoate (TMR-HTL^{SO4}) 6

1.5 General procedure A for SHTL conjugates 6

1.6 3-(3-(6-(Bis(methyl-d$_3$)amino)-3-(bis(methyl-d$_3$)iminio)-3*H*-xanthen-9-yl)-4-carboxy-*N*-(2-(2-((6-chlorohexyl)oxy)ethoxy)ethyl)benzamido)propane-1-sulfonate (TMR-d12-SHTL) 7

1.7 3-(3-(7-(Bis(methyl-d$_3$)amino)-3-(bis(methyl-d$_3$)iminio)-5,5-dimethyl-3,5-dihydrodibenzo[*b,e*]silin-10-yl)-4-carboxy-*N*-(2-(2-((6-chlorohexyl)oxy)ethoxy)ethyl)benzamido)propane-1-sulfonate (SiR-d12-HTL) 8

1.8 3-(3-(3-(Azetidin-1-ium-1-ylidene)-6-(azetidin-1-yl)-3*H*-xanthen-9-yl)-4-carboxy-*N*-(2-(2-((6-chlorohexyl)oxy)ethoxy)ethyl)benzamido)propane-1-sulfonate (JF$_{549}$-SHTL) 9

1.9 3-(3-(3-(Azetidin-1-ium-1-ylidene)-7-(azetidin-1-yl)-5,5-dimethyl-3,5-dihydrodibenzo[*b,e*]silin-10-yl)-4-carboxy-*N*-(2-(2-((6-chlorohexyl)oxy)ethoxy)ethyl)benzamido)propane-1-sulfonate (JF$_{646}$-SHTL) 10

1.10 ATTO 647N-HTL 11

1.11 ATTO 647N-SHTL 12

1.12 ATTO 647N-HTL.2 13

1.13 ATTO 647N-S$_2$HTL.2 14

1.14 NBD-HTL 15

1.15 NBD-SHTL 16

2 NMR-Spectra 17

2.1 3-(3-(6-(Bis(methyl-d$_3$)amino)-3-(bis(methyl-d$_3$)iminio)-3*H*-xanthen-9-yl)-4-carboxy-*N*-(2-(2-((6-chlorohexyl)oxy)ethoxy)ethyl)benzamido)propane-1-sulfonate (TMR-d12-SHTL) 17

2.2 3-(3-(7-(Bis(methyl-d$_3$)amino)-3-(bis(methyl-d$_3$)iminio)-5,5-dimethyl-3,5-dihydrodibenzo[*b,e*]silin-10-yl)-4-carboxy-*N*-(2-(2-((6-chlorohexyl)oxy)ethoxy)ethyl)benzamido)propane-1-sulfonate (SiR-d12-HTL) 17

2.3 *N*-(2-(2-((6-chlorohexyl)oxy)ethoxy)ethyl)-7-nitrobenzo[c][1,2,5]oxadiazol-4-amine (NBD-HTL) 18

2.4 3-((2-(2-((6-chlorohexyl)oxy)ethoxy)ethyl)(7-nitrobenzo[c][1,2,5]oxadiazol-4-yl)amino)propane-1-sulfonate (NBD-SHTL) 18

3 Computational modelling 19

3.1 Methods 19

3.2 Statistical Analysis 20

4 Supplementary Figures 22

5 Supplementary Tables 33
6 One-step reaction protocol 35
7 References 36

1 Synthesis

1.1 *tert*-Butyl (2-(2-((6-hydroxyhexyl)oxy)ethoxy)ethyl)carbamate (BocNH-HTL^{OH})

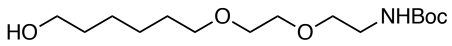

tert-Butyl (2-(2-((6-iodohexyl)oxy)ethoxy)ethyl)carbamate (**NHBoc-HTL**) (67.3 mg, 0.162 mmol, 1.0 equiv.) was dissolved in 2.5 mL of THF. Ag_2CO_3 (45.1 mg, 0.162 mmol, 1.0 equiv.) and 6 mL of 2 M NaOH were added. The reaction mixture was stirred at reflux for 3 h and subsequently stirred at 50 °C o.n before it was allowed to cool to r.t. and quenched with sat. NH_4Cl. The reaction mixture was extracted with ethyl acetate (3x). The combined organic layers were washed with water and brine, dried (Na_2SO_4) and all volatiles were removed *in vacuo* to provide the desired product (**13**) (57.9 mg, 0.190 mmol) as a yellow oil in a quantitative yield.

1H-NMR (600 MHz, $CDCl_3$): δ [ppm] = 5.11 (s, 1H), 3.61 (m, 4H), 3.53 (m, 4H), 3.44 (t, J = 6.7 Hz, 2H), 3.29 (m, 2H), 1.56 (m, 4H), 1.41 (s, 9H), 1.36 (m, 4H).

13C{1H}-NMR (150 MHz, $CDCl_3$): δ [ppm] = 156.0, 79.1, 71.3, 70.2, 70.2, 69.9, 62.6, 40.3, 32.6, 29.6, 29.4, 28.4, 25.8, 25.4.

HRMS (ESI): calc. for $C_{15}H_{32}NO_5$ $[M + H]^+$: 306.2275, found: 306.2277.

1.2 2-(6-(Dimethylamino)-3-(dimethyliminio)-3*H*-xanthen-9-yl)-4-((2-(2-((6-hydroxyhexyl)oxy)ethoxy)ethyl)carbamoyl)benzoate (TMR-HTL^{OH})

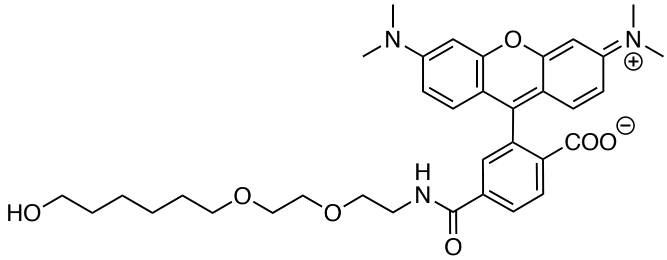

tert-Butyl (2-(2-((6-hydroxyhexyl)oxy)ethoxy)ethyl)carbamate (**BocNH-HTL^{OH}**) (10.0 mg, 32.7 μmol, 4.0 equiv.) was dissolved in 10 mL of DCM before it was cooled to 0 °C. 300 µL of TFA were added and the solution was stirred for 1 h. All volatiles were removed *in vacuo.* The residue was dissolved in DMF (150 μL) before DIPEA (200 μL) was added. TMR-NHS (4.0 mg, 7.6 μmol, 1.0 equiv.) was dissolved in DMF (150 μL) and added to the reaction mixture. The reaction was allowed to stir for 1 h and subsequently quenched by addition of 200 µL acetic acid. Preparative RP-HPLC (MeCN:H_2O+0.1% TFA = 10:90 to 90:10 over 60 minutes) provided the desired product (**TMR-HTL^{OH}**) (4.1 mg, 6.64 µmol) as pink powder in 87% yield.

1H-NMR (600 MHz, $CDCl_3$): δ [ppm] = 8.41 (d, J = 8.2 Hz, 1H), 8.22 (dd, J = 8.2, 1.8 Hz, 1H), 7.83 (d, J = 1.8 Hz, 1H), 7.17 (d, J = 9.5 Hz, 2H), 7.07 (dd, J = 9.5, 2.5 Hz, 2H), 7.00 (d, J = 2.5 Hz, 2H), 3.66 (m, 2H), 3.62 (m, 2H), 3.60 (m, 2H), 3.57 (m, 2H), 3.52 (t, J = 6.6 Hz, 2H), 3.43 (t, J = 6.6 Hz, 2H), 3.32 (s, 12H), 1.50 (m, 4H), 1.31 (m, 4H).

HRMS (ESI): calc. for $C_{35}H_{44}N_3O_7$ $[M + H]^+$: 618.3174, found: 618.3182.

1.3 2-(6-(Dimethylamino)-3-(dimethyliminio)-3H-xanthen-9-yl)-4-((2-(2-((6-(phosphonooxy)hexyl)oxy)ethoxy)ethyl)carbamoyl)benzoate (TMR-HTL^{PO4})

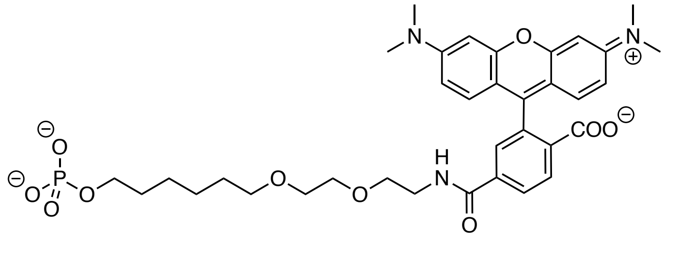

Phosphoroxychloride (1.2 mg, 7.83 µmol, 5.0 equiv.) was dissolved in DCM (50 μL) and cooled to 0 °C. **TMR-HTL^{OH}** (1.0 mg, 1.62 µmol, 1.0 equiv.) was also dissolved in DCM (50 μL) and DIPEA (2.1 mg, 16.2 μmol, 10.0 equiv.) was added. The mixture was added dropwise to the solution of phosphoroxychloride and the reaction was stirred o.n. at r.t. The reaction was quenched with 1 mL of water and all volatiles were removed *in vacuo.* Preparative RP-HPLC (MeCN:H_2O+0.1% TFA = 10:90 to 95:05 over 60 minutes) provided the desired product (**TMR-HTL^{PO4}**) (194 nmol) as pink powder in 12% yield.

HRMS (ESI): calc. for $C_{35}H_{45}N_3O_{10}P$ $[M + H]^+$: 698.2837, found: 698.2843.

1.4 2-(6-(Dimethylamino)-3-(dimethyliminio)-3H-xanthen-9-yl)-4-((2-(2-((6-(sulfooxy)hexyl)oxy)ethoxy)ethyl)carbamoyl)benzoate (TMR-HTL^{SO4})

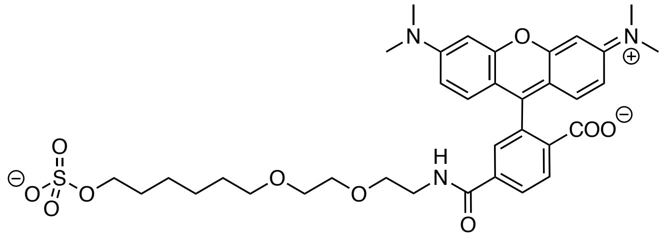

Sulfurylchloride (0.7 mg, 5.0 µmol, 5.0 equiv.) was dissolved in DCM (50 µL) and cooled to 0 °C. **TMR-HTL^{OH}** (0.6 mg, 1.0 µmol, 1.0 equiv.) was also dissolved in DCM (50 µL) and DIPEA (1.3 mg, 10.0 µmol, 10.0 equiv.) was added. The mixture was added dropwise to the solution of sulfurylchloride and the reaction was stirred o.n. at r.t. The reaction was quenched with 1 mL of water and all volatiles were removed *in vacuo.* Preparative RP-HPLC (MeCN:H_2O+0.1% TFA = 10:90 to 95:05 over 60 minutes) provided the desired product (**TMR-HTL^{SO4}**) (30.1 nmol) as pink powder in 3% yield.

HRMS (ESI): calc. for $C_{35}H_{44}N_3O_{10}S$ $[M + H]^+$: 698.2742, found: 698.2713

1.5 General procedure A for SHTL conjugates

A 5 nmol aliquot of HTL conjugated fluorophore was dissolved in 4 µL *tert*-butoxide (100 mM in DMSO) and 1 µL of 35 °C warm 1,3-propane sultone was added. After the reaction was complete (monitored by LCMS, which takes around 5 min), the reaction was quenched with 5 µL PBS and used without further purification.

1.6 3-(3-(6-(Bis(methyl-d3)amino)-3-(bis(methyl-d3)iminio)-3*H*-xanthen-9-yl)-4-carboxy-*N*-(2-(2-((6-chlorohexyl)oxy)ethoxy)ethyl)benzamido)propane-1-sulfonate (TMR-d12-SHTL)

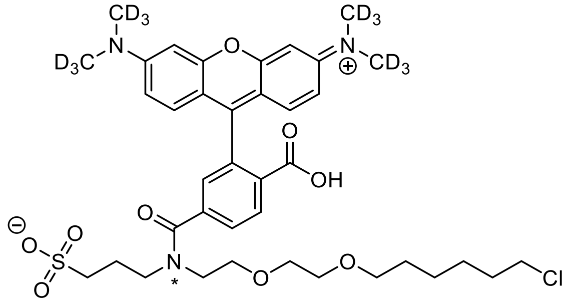

TMR-d12-SHTL was prepared according to general procedure A with TMR-d12-HTL or by the following procedure:

TMR-d12-HTL (1.88 mg, 2.90 µmol, 1.0 equiv.) was dissolved in DMF, before NaH 60% in mineral oil (2.32 mg, 58.0 µmol, 20.0 equiv.) and 1,3-propane sultone (1.77 mg, 14.5 µmol, 5.0 equiv.) were added. The reaction was stirred for 1 h at rt, before it was quenched by addition of glacial HOAc (50 µL). The reaction mixture was subjected to RP-HPLC to obtain 2.10 mg (2.73 µmol) of the desired compound as a blue powder in 94% yield. NMR spectra are reported for *cis-/trans*-amide isomers.

^{1}H NMR (600 MHz, DMSO-d$_6$) δ [ppm] = 8.25 (q, J = 7.7 Hz, 2H), 7.79 (d, J = 7.9 Hz, 1H), 7.73 (d, J = 7.9 Hz, 1H), 7.52 (s, 1H), 7.38 (s, 1H), 7.17 (s, 4H), 7.10 (s, 4H), 6.95 (s, 4H), 3.58 (t, J = 8.5 Hz, 8H), 3.51 (q, J = 6.6 Hz, 10H), 3.46 (d, J = 3.3 Hz, 2H), 3.20 (s, 2H), 3.14 (t, J = 6.5 Hz, 2H), 2.55 (s, 4H), 2.43 (t, J = 7.3 Hz, 2H), 2.26 (t, J = 7.0 Hz, 2H), 1.88 (t, J = 7.1 Hz, 2H), 1.72 (t, J = 6.8 Hz, 2H), 1.65 (m, J = 7.7 Hz, 4H), 1.42 (q, J = 6.8 Hz, 2H), 1.30 (m, J = 8.0 Hz, 8H), 1.17 (t, J = 7.2 Hz, 2H).

HRMS (ESI): calc. for $C_{38}H_{37}D_{12}ClN_3O_9S$ $[M]^+$: 770.3626, found: 770.3632.

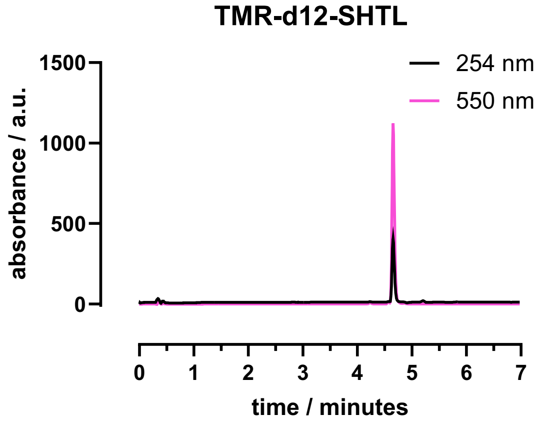

1.7 3-(3-(7-(Bis(methyl-d3)amino)-3-(bis(methyl-d3)iminio)-5,5-dimethyl-3,5-dihydrodibenzo[*b,e*]silin-10-yl)-4-carboxy-*N*-(2-(2-((6-chlorohexyl)oxy)ethoxy)ethyl)benzamido)propane-1-sulfonate (SiR-d12-HTL)

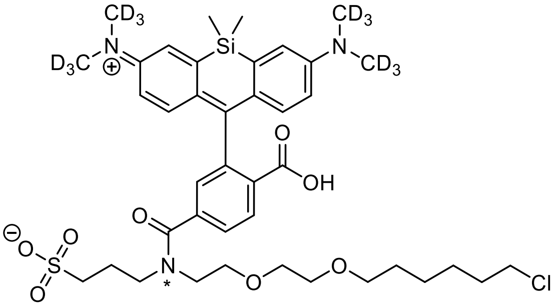

SiR-d12-SHTL was prepared according to general procedure A with SiR-d12-HTL or by the following procedure:

SiR-d12-HTL (2.0 mg, 2.90 μmol, 1.0 equiv.) was dissolved in 500 μL DMF, before NaH 60% in mineral oil (2.32 mg, 58.0 μmol, 20.0 equiv.) and 1,3-propane sultone (1.77 mg, 14.5 μmol, 5.0 equiv.) were added. The reaction was stirred for 1 h at rt, before it was quenched by addition of glacial HOAc (50 μL). The reaction mixture was subjected to RP-HPLC to obtain 2.14 mg (2.64 µmol) of the desired compound as a blue powder in 91% yield. NMR spectra are reported for *cis*-/*trans*-amide isomers.

^{1}H NMR (600 MHz, D_2O) δ [ppm] = 8.18 (dd, *J* = 8.0, 15.1 Hz, 1H), 8.00 (t, *J* = 2.8 Hz, 3H), 7.83 (d, *J* = 8.0 Hz, 1H), 7.79 (d, *J* = 8.0 Hz, 1H), 7.69 (s, 1H), 7.56 (dd, *J* = 2.5, 8.8 Hz, 1H), 7.49 (d, *J* = 8.8 Hz, 1H), 7.40 (s, 1H), 7.32 (d, *J* = 8.9 Hz, 1H), 7.22 (d, *J* = 8.9 Hz, 1H), 3.79-3.71 (m, 1H), 3.66-3.60 (m, 1H), 3.53 (bs, 1H), 3.40 (t, *J* = 7.7 Hz, 1H), 3.37 (t, *J* = 6.6 Hz, 1H), 3.31-3.25 (m, 1H), 2.95 (t, *J* = 7.6 Hz, 1H), 2.87 (t, *J* = 6.4 Hz, 1H), 2.48 (t, *J* = 7.5 Hz, 1H), 2.09 (p, *J* = 7.4 Hz, 1H), 1.85 (p, *J* = 7.3 Hz, 1H), 1.42 (p, *J* = 7.3 Hz, 1H), 1.34 (p, *J* = 7.2 Hz, 1H), 1.17 (p, *J* = 7.2 Hz, 1H), 1.0-0.95 (m, 1H), 0.91 (p, *J* = 7.6 Hz, 1H), 0.84 (p, *J* = 7.6 Hz, 1H), 0.77 (s, 1H), 0.65 (s, 1H), 0.63 (s, 1H).

HRMS (ESI): calc. for $C_{40}H_{43}D_{12}ClN_3O_8SSi$ [M]$^+$: 812.3915, found: 812.3915.

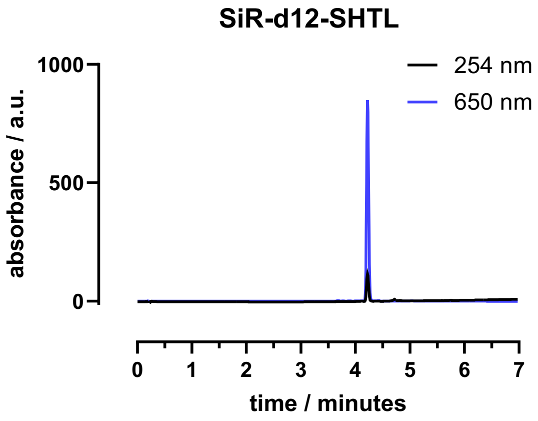

1.8 3-(3-(3-(Azetidin-1-ium-1-ylidene)-6-(azetidin-1-yl)-3*H*-xanthen-9-yl)-4-carboxy-*N*-(2-(2-((6-chlorohexyl)oxy)ethoxy)ethyl)benzamido)propane-1-sulfonate (JF$_{549}$-SHTL)

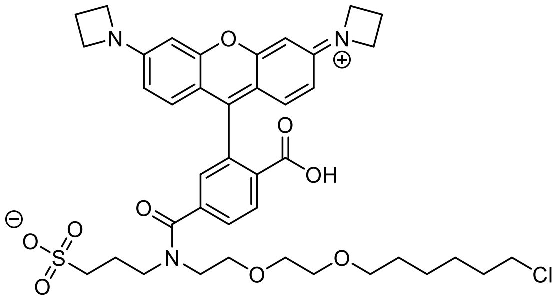

JF$_{549}$-SHTL was prepared according to general procedure A with JF$_{549}$-HTL and used without further purification.

HRMS (ESI): calc. for $C_{40}H_{49}ClN_3O_9S$ $[M]^+$: 782.2873, found: 782.2873.

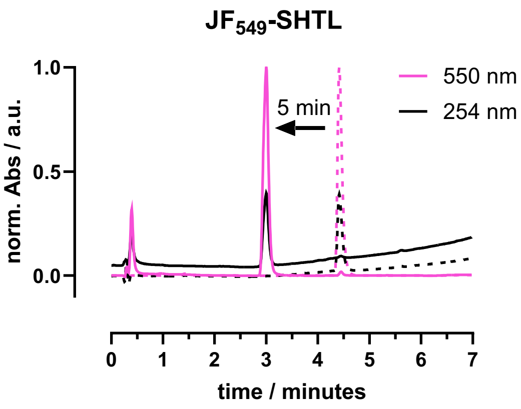

1.9 3-(3-(3-(Azetidin-1-ium-1-ylidene)-7-(azetidin-1-yl)-5,5-dimethyl-3,5-dihydrodibenzo[*b,e*]silin-10-yl)-4-carboxy-*N*-(2-(2-((6-chlorohexyl)oxy)ethoxy)ethyl)benzamido)propane-1-sulfonate (JF$_{646}$-SHTL)

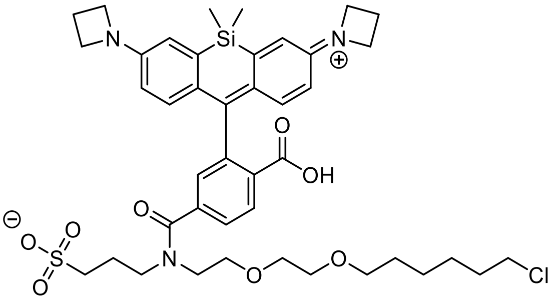

JF$_{646}$-SHTL was prepared according to general procedure A with JF$_{646}$-HTL and used without further purification.

HRMS (ESI): calc. for $C_{42}H_{55}ClN_3O_8SSi$ $[M]^+$: 824.3162, found: 824.3162.

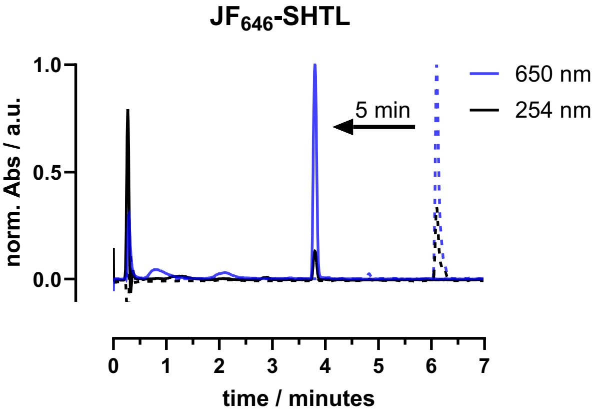

1.10 ATTO 647N-HTL

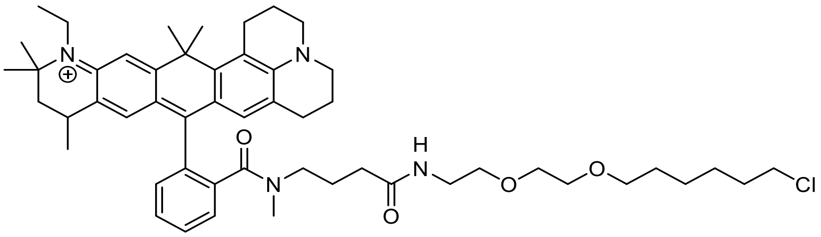

ATTO 647N-NHS (0.5 mg, 673 nmol, 1.0 equiv.) was dissolved in 500 µL DMSO, before DIPEA (0.5 μL, 2.69 μmol, 4.0 equiv.) and 1.2 equiv. HTL-NH_2 (0.3 mg, 1.35 μmol, 1.2 equiv.) were added. The mixture was vortexed again and allowed to incubate for 60 min before it was quenched by addition of glacial HOAc (50 μL). HPLC (MeCN:H_2O+0.1% TFA = 30:70 to 90:10 over 46 minutes) provided the desired compound (612 nmol, 91%) as blue powder after lyophilization.

HRMS (ESI): calc. for $C_{52}H_{72}ClN_4O_4$ $[M]^+$: 851.5237, found: 851.5233.

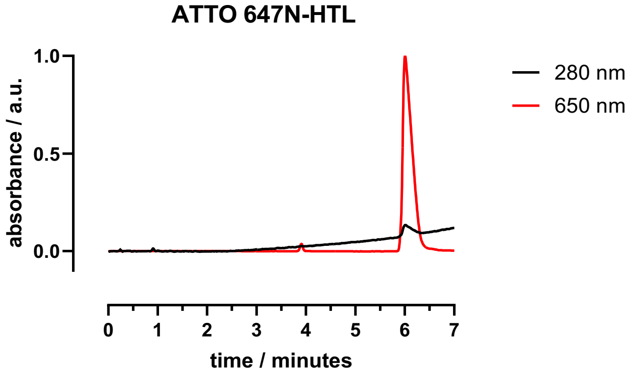

1.11 ATTO 647N-SHTL

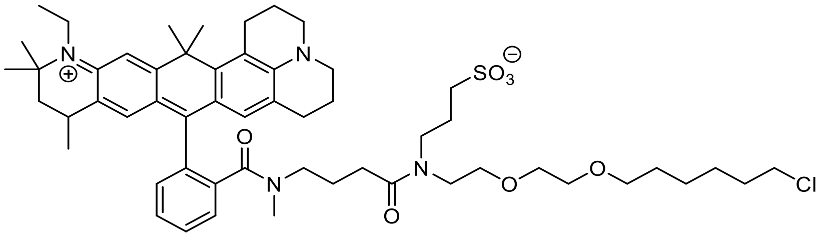

ATTO 647N-HTL (61.0 μg, 72 nmol, 1.0 equiv.) was dissolved in 200 μL DMF, before NaH 60% in mineral oil (57.6 μg, 1.44 μmol, 20.0 equiv.) and 1,3-propane sultone (44 μg, 360 nmol, 5.0 equiv.) was added. The reaction was briefly sonicated, before it was stirred for 6 h at rt. The mixture was quenched by addition of glacial HOAc (50 μL) and subjected to RP-HPLC (MeCN:H_2O+0.1% TFA = 30:70 to 90:10 over 46 minutes) to obtain 6.8 μg (7 nmol) of the desired compound as a blue powder in 10% yield, with starting material (40 nmol, 56%) recovered.

HRMS (ESI): calc. for $C_{55}H_{78}ClN_4O_7S$ [M + H]$^{2+}$: 487.2674, found: 487.2669.

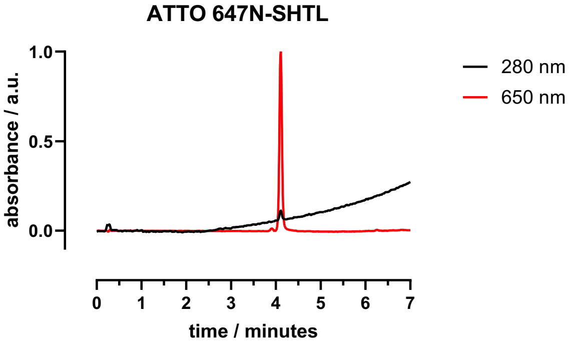

1.12 ATTO 647N-HTL.2

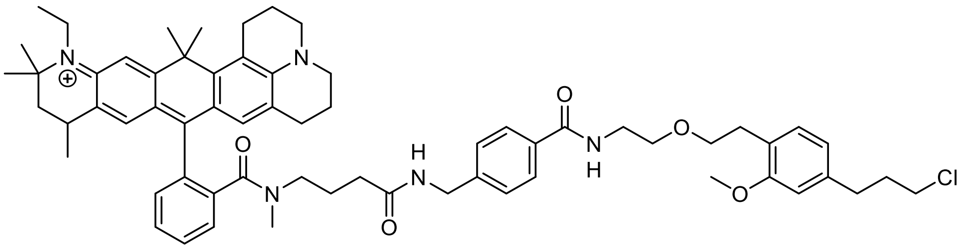

ATTO 647N-NHS (0.5 mg, 673 nmol, 1.0 equiv.) was dissolved in 500 µL DMSO, before DIPEA (0.5 *µ*L, 2.69 *µ*mol, 4.0 equiv.) and HTL.2-NH_2 (0.5 mg, 1.35 *µ*mol, 1.2 equiv.) were added. The mixture was vortexed again and allowed to incubate for 60 min before it was quenched by addition of glacial HOAc (50 *µ*L). HPLC (MeCN:H_2O+0.1% TFA = 30:70 to 90:10 over 46 minutes) provided the desired compound (599 nmol, 89%) as blue powder after lyophilization.

HRMS (ESI): calc. for $C_{64}H_{79}ClN_5O_5$ $[M + H]^{2+}$: 516.7919, found: 516.7909.

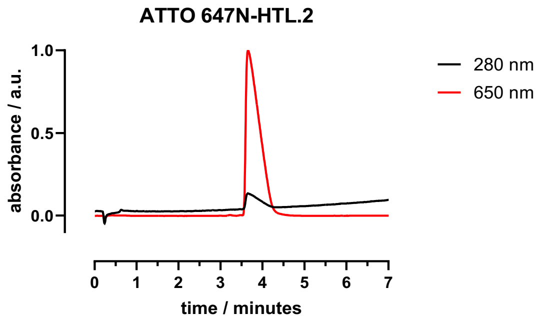

1.13 ATTO 647N-S_2HTL.2

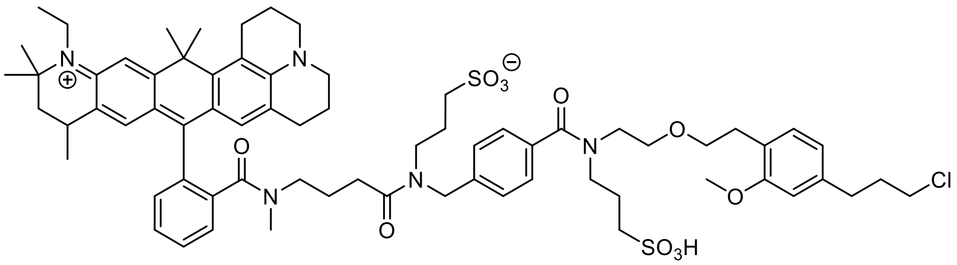

ATTO 647N-HTL.2 (145 µg, 170 nmol, 1.0 equiv.) was dissolved in 200 µL DMF, before NaH 60% in mineral oil (136 µg, 3.4 µmol, 20.0 equiv.) and 1,3-propane sultone (104 µg, 850 nmol, 5.0 equiv.) were added. The reaction was briefly sonicated, before it was stirred for 6 h at rt. The mixture was quenched by addition of glacial HOAc (50 µL) and subjected to RP-HPLC (MeCN:H_2O+0.1% TFA = 10:90 to 90:10 over 46 minutes) to obtain 29 µg (23 nmol) of the desired compound as a blue powder in 14% yield, with starting material (68 nmol, 40%) recovered.

HRMS (ESI): calc. for $C_{70}H_{91}ClN_5O_{11}S_2$ $[M + H]^{2+}$: 638.7956, found: 638.7967.

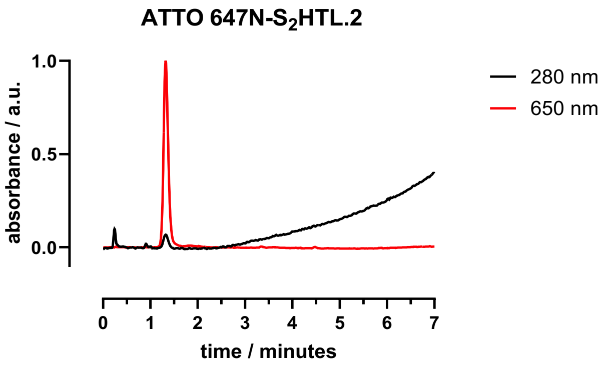

1.14 NBD-HTL

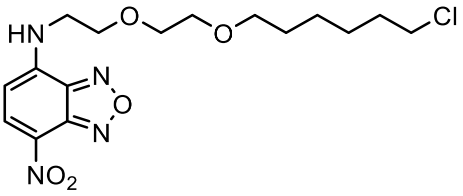

4-chloro-7-nitrobenzo[c][1,2,5]oxadiazole (10 mg, 50 µmol, 1.0 equiv.) was dissolved in 1 mL of anhydrous EtOH, before Et_3N (13.57 *µ*L, 150 µmol, 3.0 equiv.) was added followed by HTL-NH_2 (15.0 mg, 60 µmol, 1.2 equiv.). The reaction mixture was stirred for 4 h before all volatiles were removed *in vacuo*. The residue was purified by HPLC (MeCN:H_2O+0.1% TFA = 10:90 to 90:10 over 60 minutes) and 15.4 mg of the desired compound as an orange powder in 80% yield was obtained after lyophilization.

^{1}H-NMR (600 MHz, MeOD-d_4): δ [ppm] = 8.56 (d, *J* = 8.9 Hz, 1H), 6.48 (d, *J* = 8.9 Hz, 1H), 3.85 (t, *J* = 5.2 Hz, 2H), 3.79 (s, 1H), 3.69 (m, 2H), 3.62 (m, 2H), 3.56 (t, *J* = 6.6 Hz, 2H), 3.48 (t, *J*= 6.6 Hz, 2H), 1.75 (q, *J* = 7.1 Hz, 2H), 1.56 (q, *J* = 7.4 Hz, 2H), 1.44 (q, *J* = 7.7 Hz 2H), 1.35 (m, 2H).

HRMS (ESI): calc. for $C_{16}H_{23}ClN_4O_5Na$ [M + Na]$^+$: 409.1249, found: 409.1274.

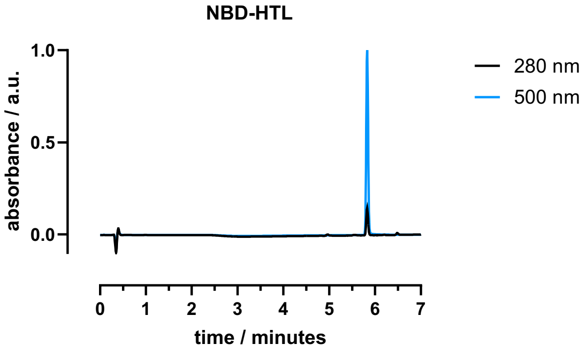

1.15 NBD-SHTL

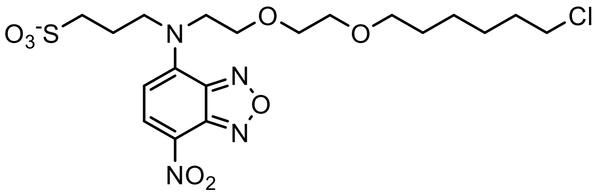

In an Eppendorf tube was added the NBD-HTL (1.3 mg, 3.4 µmol, 1 eq.) followed by the NaH (1.63 mg, 68.20 µmol, 20 eq.) dissolved in DMF and 1,3-propanesultone (2 mg, 17.5 µmol, 5 eq.). The experiments were carried out at rt. The reaction was followed by LC-MS until 100% conversion was observed. The product was purified by HLPC (MeCN:H_2O+0.1% TFA = 10:90 over 60 min) and 0.95 mg of the desired compound as an orange powder in 55% yield was obtained after lyophilization.

^{1}H-NMR (600 MHz, MeOD-d_4): δ [ppm] = 8.55 (d, J = 9.2 Hz, 1H), 6.64 (d, J = 9.3 Hz, 1H), 4.37 (s, 2H), 4.19 (s, 2H), 3.91 (t, J = 5.3 Hz, 2H), 3.64 (m, 2H), 3.56 (m, 4H), 3.41 (t, J = 5.3 Hz, 2H), 2.98 (t, J = 7.2 Hz, 2H), 2.30 (q, *J* = 7.8 Hz, 2H), 1.76 (q, *J* = 7.3 Hz, 2H), 1.51 (m, 2H), 1.45 (q, *J* = 7.6 Hz, 2H), 1.33 (m, 2H).

HRMS (ESI): calc. for $C_{19}H_{29}ClN_4O_8SNa$ [M + H + Na]$^+$: 531.1287, found: 531.1312.

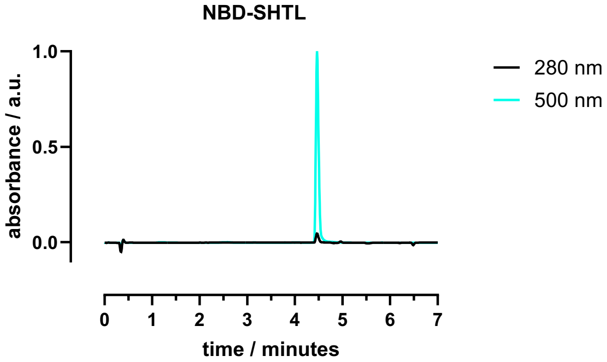

2 NMR-Spectra

2.1 3-(3-(6-(Bis(methyl-d3)amino)-3-(bis(methyl-d3)iminio)-3*H*-xanthen-9-yl)-4-carboxy-*N*-(2-(2-((6-chlorohexyl)oxy)ethoxy)ethyl)benzamido)propane-1-sulfonate (TMR-d12-SHTL)

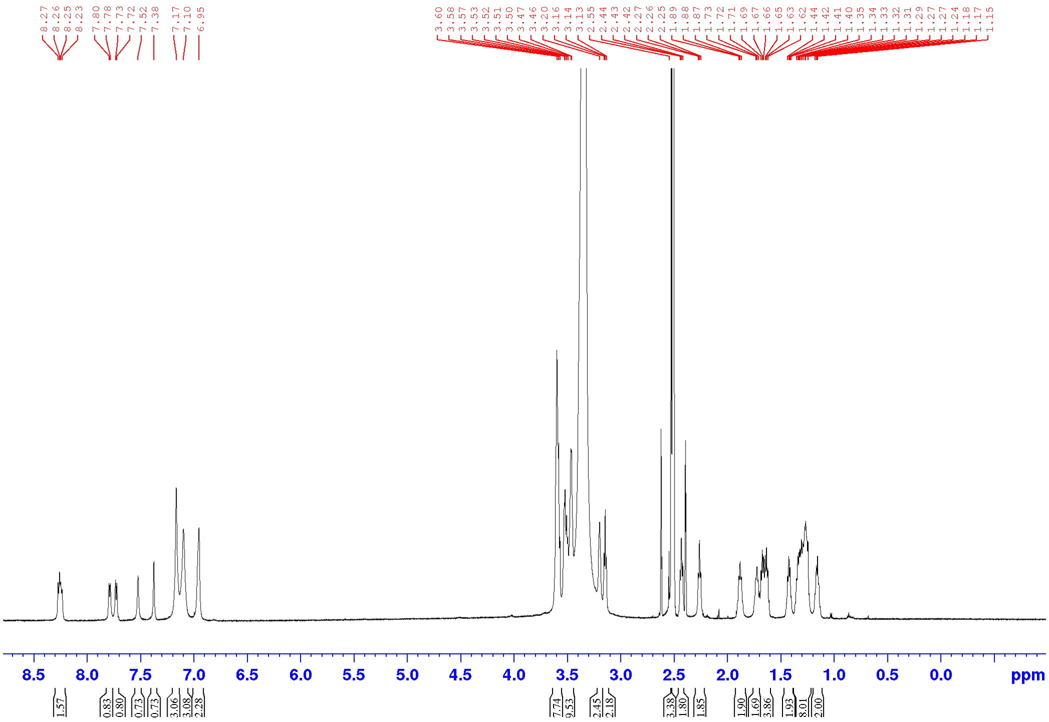

2.2 3-(3-(7-(Bis(methyl-d3)amino)-3-(bis(methyl-d3)iminio)-5,5-dimethyl-3,5-dihydrodibenzo[*b,e*]silin-10-yl)-4-carboxy-*N*-(2-(2-((6-chlorohexyl)oxy)ethoxy)ethyl)benzamido)propane-1-sulfonate (SiR-d12-HTL)

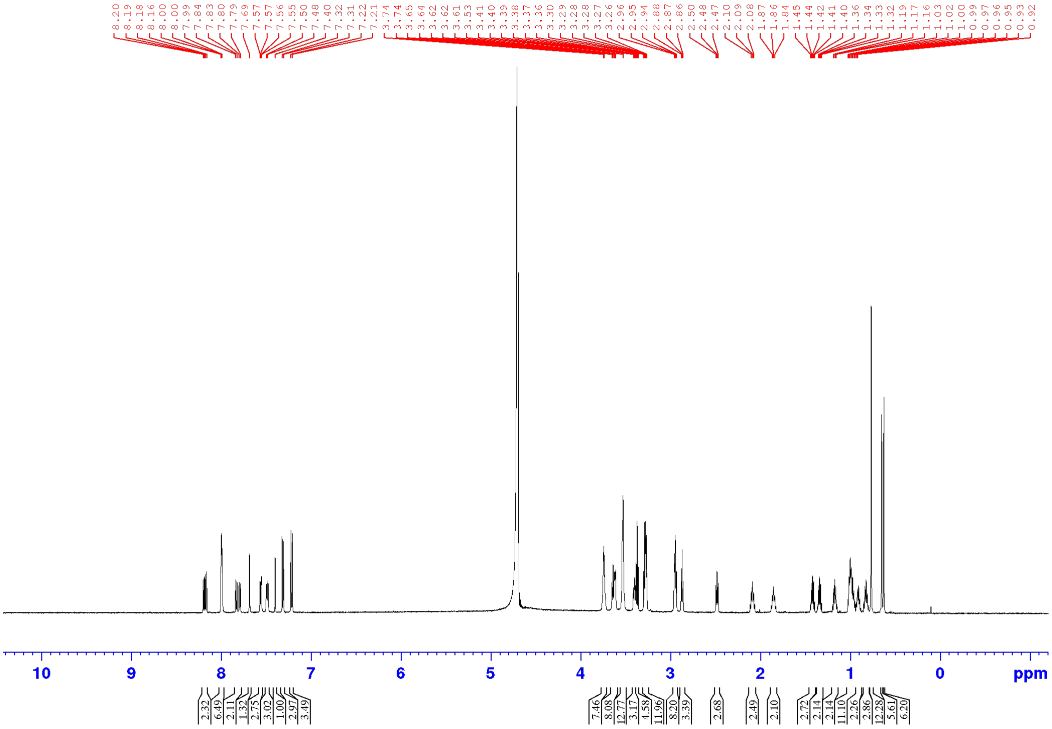

2.3 *N*-(2-(2-((6-chlorohexyl)oxy)ethoxy)ethyl)-7-nitrobenzo[c][1,2,5]oxadiazol-4-amine (NBD-HTL)

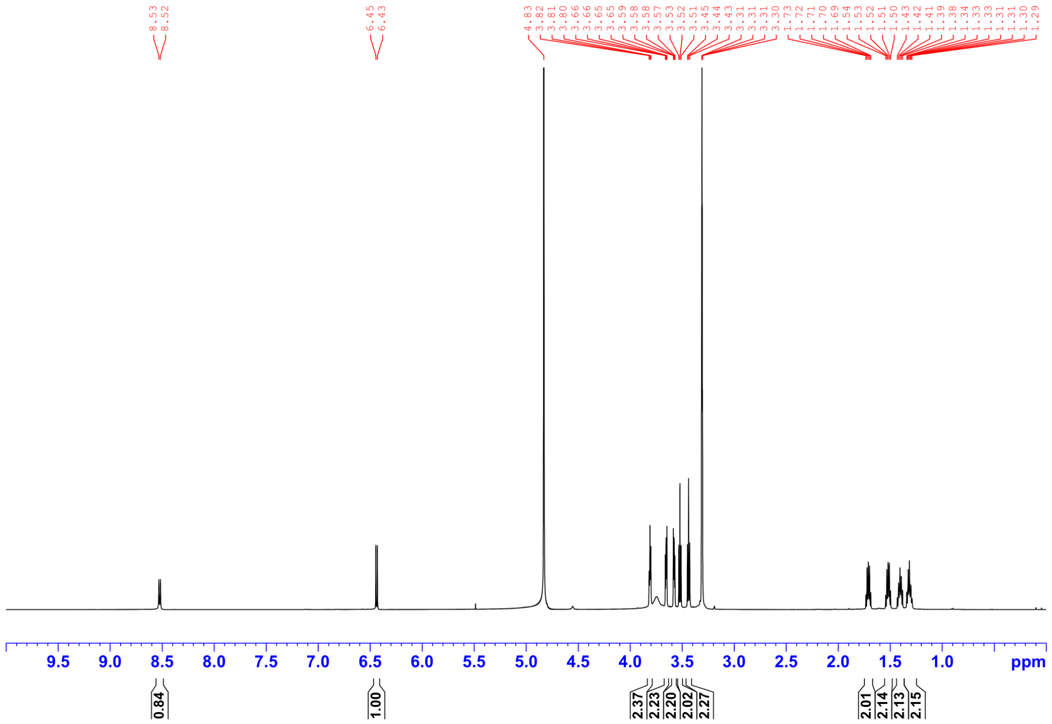

2.4 3-((2-(2-((6-chlorohexyl)oxy)ethoxy)ethyl)(7-nitrobenzo[c][1,2,5]oxadiazol-4-yl)amino)propane-1-sulfonate (NBD-SHTL)

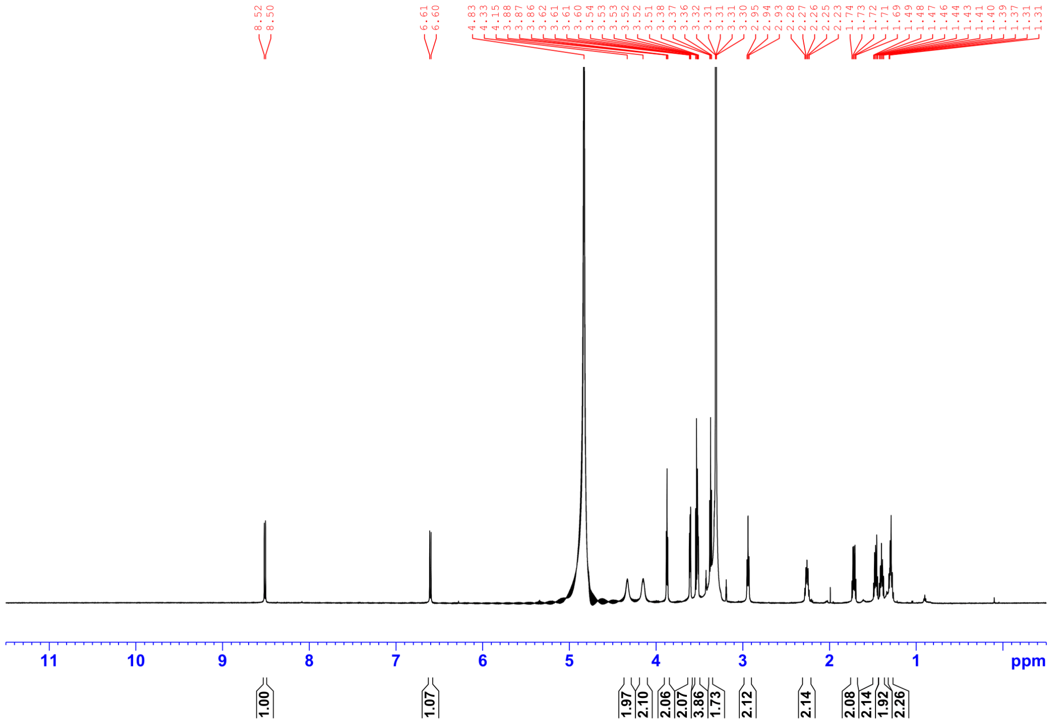

3 Computational modelling

3.1 Methods

In order to investigate the theoretical plausibility of a sulfonated HTL (SHTL) compound as a ligand with comparable or preferably increased affinity towards the HTP receptor, computational docking was performed.

To this end, the isolated ligand geometries of the dye-HTL and dye-SHTL conjugates (**Supplementary Figure 2**) were first obtained from a SMILES string, and pre-optimized using OpenBabel heuristics in combination with the MMFF94 force field (conjugated gradient algorithm, 3D conformer generation setting „best“).[1–5] Subsequently, the structures were geometry optimized using the ORCA6 quantum chemistry suite.[6] Here, the ωB97X-D3BJ/def2-TZVPP method was used with the default integration grid settings and both TightSCF and TightOPT convergence criteria. The RIJCOSX system was employed throughout with automatic auxiliary basis set selection, and to model solvation effects, the SMD method was used for water.[7,8] After geometry convergence, the stability of the minimum was validated by calculating the vibrational frequencies using the same method, affording exclusively positive vibrational frequencies. The receptor was obtained from the RCSB database, whereas the apo structure of the HaloTag protein was taken from PDB-5UY1.[9] Using the ChimeraX Dock Prep routine, the receptor was prepared by deleting solvent, ions, and non-standard residue entities, building incomplete residues from the Dunbrack rotamer library, adding hydrogens, and adjusting charges for physiological conditions.[10,11]

All docking experiments were performed using the GNINA 1.1 docking program, whereas the empirical scoring function „Vinardo“ was employed, in tandem with the CNN model „general_default2018“, used for re-scoring of the final poses.[12,13] All experiments used an exhaustiveness of 128 to search for minima, whereas a total of 50 output poses was requested. To establish reproducibility, the random seed was set to 42. The search box was defined using the full receptor geometry, whereas the autobox method was used to fully enclose the receptor including 5.0 Å padding on all sides, and allowing for automatic box extension. Both ligands were docked to the receptor using the standard docking routine, affording models for the initial non-covalent association, as well as using the covalent docking approach implemented in GNINA. In the latter, the internal binding heuristics were used to simulate a covalent bond between the carboxylate of residue ASP106 and the terminal carbon of HTL and SHTL, respectively. Before covalent docking, the terminal chloride was exchanged for hydrogen, and the geometry of the ligand was re-optimized using the aforementioned DFT geometry optimization settings, but exclusively permitting hydrogen atom movement. To allow for comprehensive sampling of the conformational space, in the covalent docking, the residue ASP106 was granted torsional flexibility.

The ensemble score distributions of both ligands for both docking experiments are depicted in **Supplementary Figure 3**. It should be noted that all presented docking scores are not to be interpreted thermodynamically, but rather as descriptive parameters for the relative behaviour of both ligands. The empirical scoring function is a well-established and robust tool for screening, employing systematic treatment of

different energetic terms, whereas the CNN re-scoring function is a convolutional neural network trained to estimate affinity from complex and non-linear features, implicitly encoding solvent exposure, cooperativity and complex interaction patters that are beyond the reach of empirical scoring functions.

Selection of the best global binding poses was guided by a maximized CNN affinity score, the best poses for both ligands are depicted in **Supplementary Figure 4**. To further investigate the total ensemble of binding modes and compare both pose-score correlation and structural diversity, a principal component analysis (PCA) of all atomic cartesian coordinates for each ligand pose was performed. The first two principal components (selected by highest explained variance) are shown in **Supplementary Figure 5**.

A comprehensive study of the structural stability of the afforded complexes could be complemented by free energy perturbative methods or MM-PB/GBSA calculations on the non-covalent ensembles, as well as molecular dynamics simulations and subsequent stability evaluations for the covalently conjugated constructs.

3.2 Statistical Analysis

To compare the ligand behaviour for both dye-HTL and dye-SHTL conjugates, several statistical tests were performed on the presented data. All tests were calculated separately for (i) the *non-covalent* and (ii) the *covalent* docking ensembles, each evaluated with the two scoring modes *Empirical* and *CNN*. For each combination of ensembles and scores, two separate questions were addressed. First, the correlation of the estimated affinity scores were tested for correlation with the principal conformational variation (PC1 + PC2) of the ligand geometries, which was assessed by multivariate least-squares regression of the affinity scores against the first two principal components. Second, the ensemble affinity scores were compared for both ligands to test for statistically significant deviations. To this end, a two-sided Mann-Whitney U test was employed, affording a non-parametric comparison of the ligand ensembles.

The results for the correlation analysis are presented in **Supplementary Supplementary Table *1*** and **3**, whereas the Mann-Whitney U test is shown in **Supplementary Supplementary Table *2*** and **4**.

The CNN re-scoring revealed strong and highly significant correlations between ligand conformational variation and estimated affinity for both ligands in the non-covalent ensemble (R = 0.92 for TMR-SHTL; R = 0.46 for TMR-HTL) and retains moderate correlation in the covalent ensembles. The empirical scoring shows weak to moderate correlations throughout.

For the non-covalent ensemble, the CNN affinity scores of TMR-SHTL are shifted significantly towards more favorable values than those of TMR-HTL (U = 790, p = 0.0015), whereas the empirical scoring revealed no significant difference (U = 1375, p = 0.39). In the covalent ensembles, both scoring functions indicated a pronounced score increase for TMR-SHTL (CNN $p < 10^{-13}$; Empirical $p < 10^{-8}$).

These results collectively demonstrate that TMR-SHTL is expected to be accommodated at least as well as TMR-HTL, and, under the more expressive CNN model, consistently exhibits superior predicted binding across both binding protocols.

For the NBD conjugates, in the non-covalent ensemble, CNN re-scoring showed a moderate and statistically significant correlation with conformational variation for NBD-SHTL ($R = 0.45$, $p = 0.001$), whereas NBD-HTL exhibited weaker but still significant correlation ($R = 0.30$, $p = 0.037$). In contrast, empirical scoring yielded only weak and non-significant trends for both ligands. In the covalent ensembles, the empirical scoring showed the strongest overall correlation ($R = 0.66$ for NBD-HTL, $R = 0.38$ for NBD-SHTL), while CNN correlations were weaker but still significant for NBD-SHTL ($R = 0.37$, $p = 0.0096$).

The Mann-Whitney U tests resolve a clear and highly significant shift toward more favorable scores for NBD-SHTL compared to NBD-HTL. In the covalent ensembles, this separation is even more pronounced for both scoring methods.

Taken together, these findings indicate that SHTL substrates are predicted to bind at least as well as HTL substrates. The CNN re-scoring model consistently identifies SHTL as the stronger binder in both non-covalent and covalent ensembles, mirroring the same trends for TMR and NBD, reinforcing HTP’s general tolerance for general sulfonation of dye-HTL substrates.

4 Supplementary Figures

A

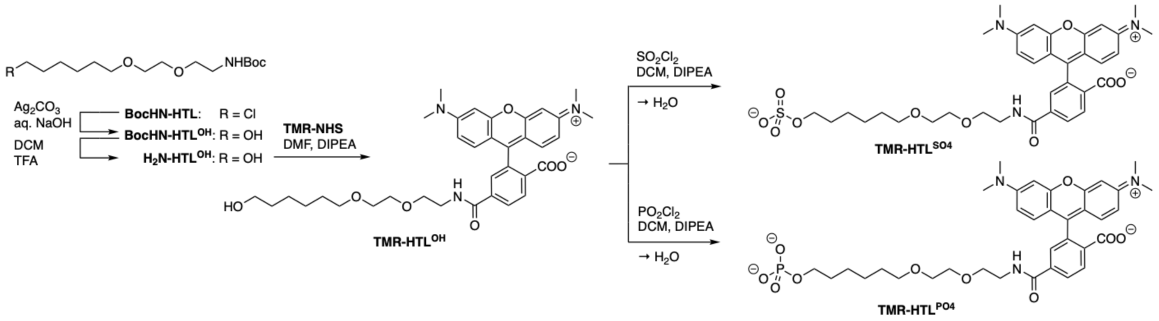

B

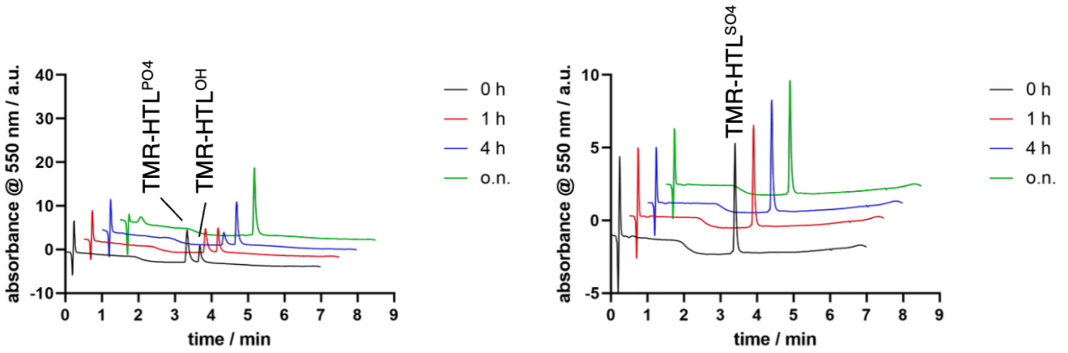

C

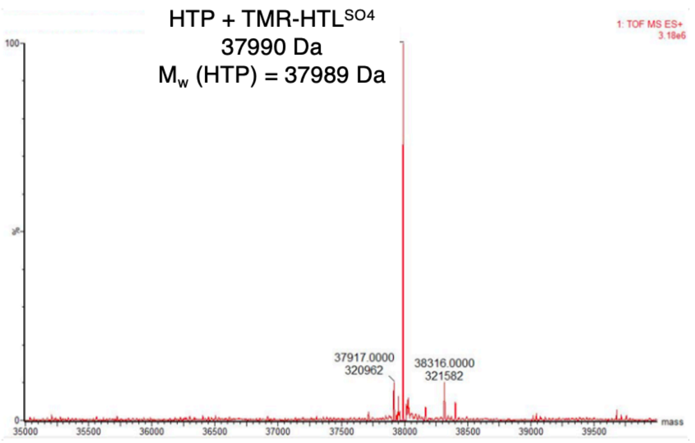

Supplementary Figure 1: A sulfonated and phosphonated HTL does not label the HTP. (A) Synthesis of **TMR-HTL^{OH}**, **TMR-HTL^{SO4}** and **TMR-HTL^{PO4}** by displacement of the chloride atom with a hydroxy group using silver carbonate, in situ deprotection of the Boc group and amide bond formation using **TMR-NHS**. The terminal charges are installed by using either sulfurylchloride or phosphooxychloride, followed by quenching the reaction with water. **B) TMR-HTL^{SO4}**, but not **TMR-HTL^{PO4}**, is stable in PBS over hours as assessed by LCMS analysis. **C**) Recombinant HTP incubated **TMR-HTL^{SO4}** is not covalently labelled, as assessed by full protein mass spectrometry. n=1.

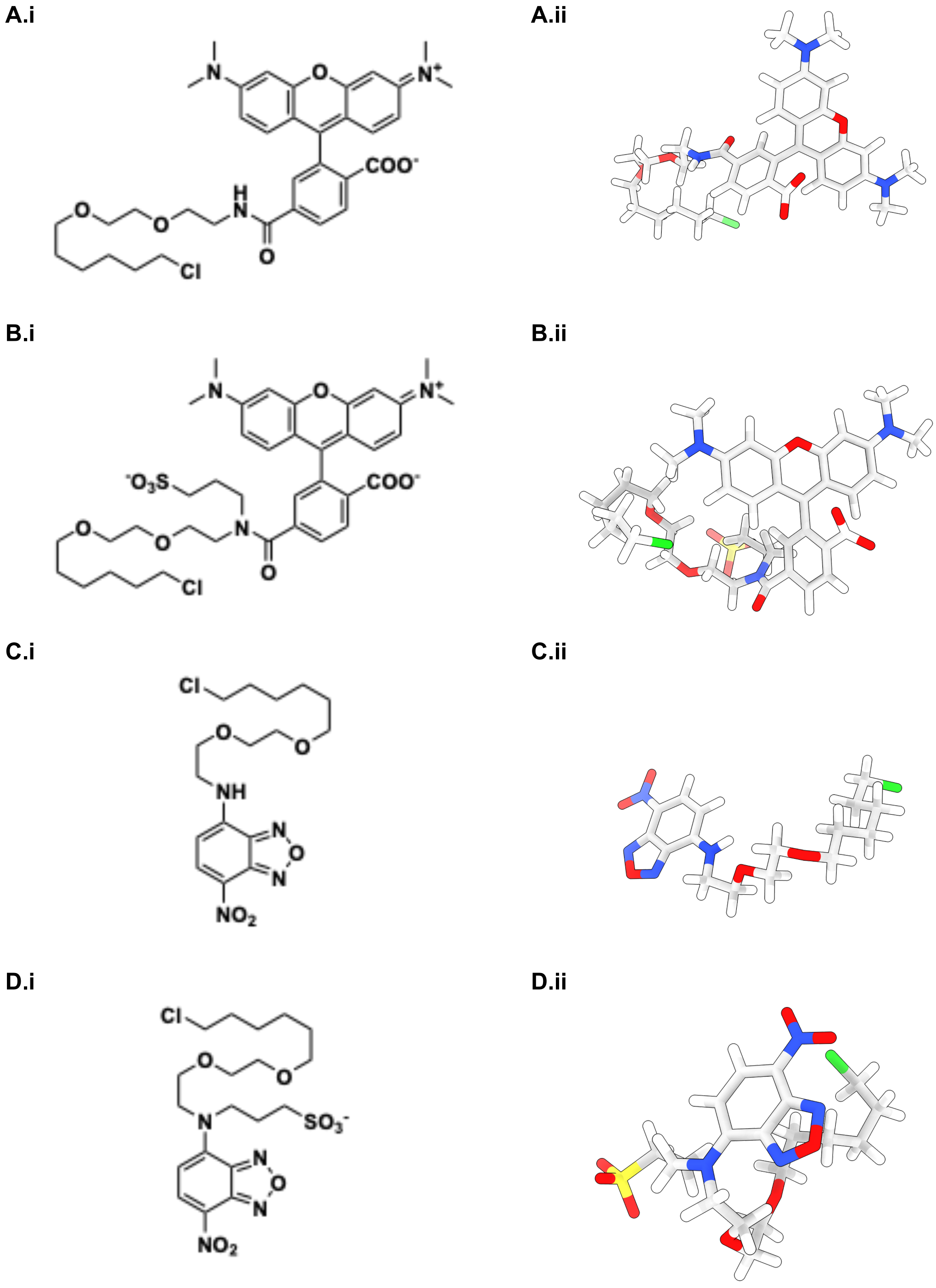

Supplementary Figure 2: Topology and optimized geometry of the studied ligands. The molecular topology of TMR-HTL **(A)** and TMR-SHTL **(B)**, as well as NBD-HTL (**C**) and NBD-SHTL (**D**) is depicted in **(i)**, whereas **(ii)** shows the optimized geometries using ωB97X-D3BJ/def2-TZVPP[SMD(water)].

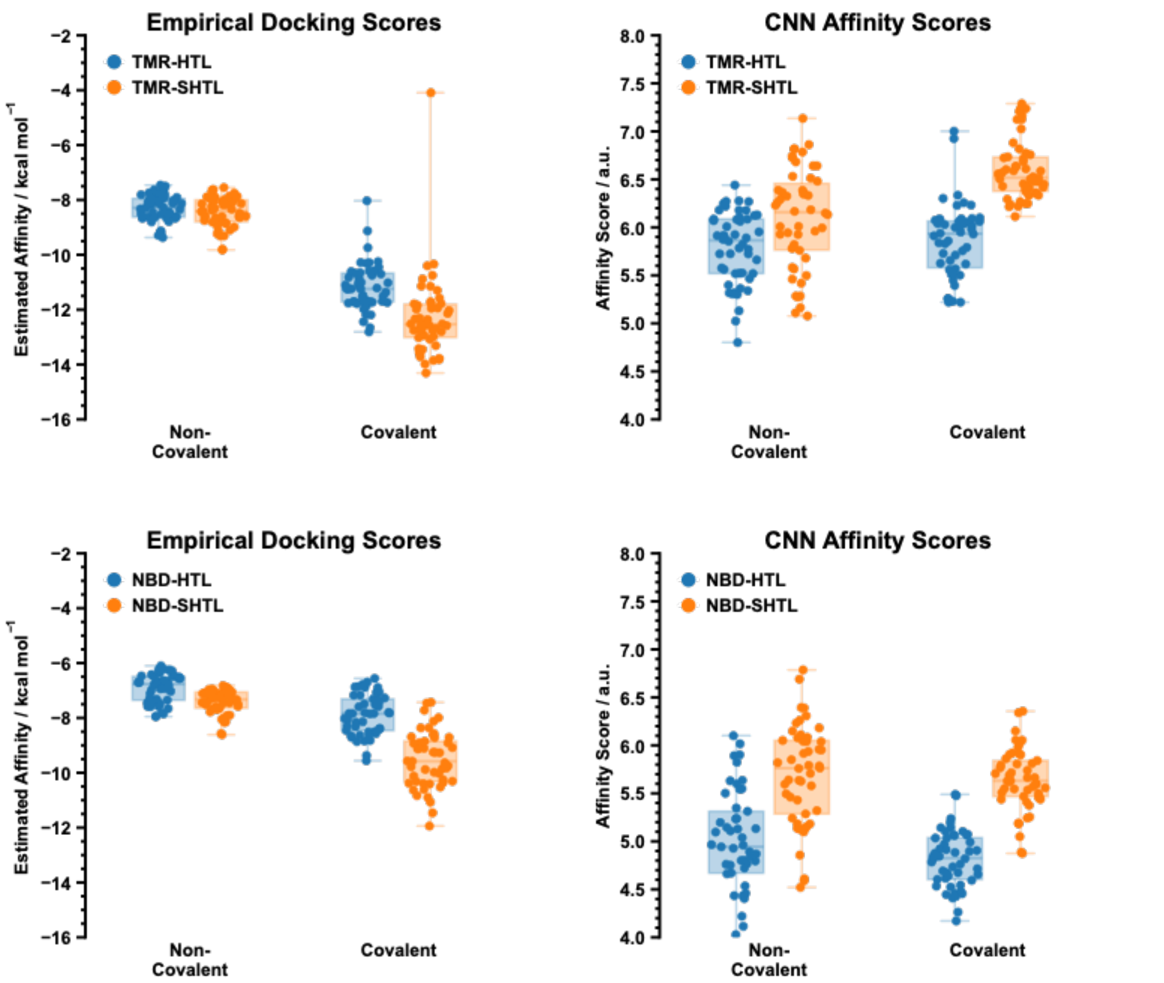

Supplementary Figure 3: Score Distributions. Empirical affinities for all poses for all ensembles of ligands, as obtained using the Vinardo scoring function. For each ensemble, all poses were re-scored using the GNINA CNN re-scoring model. (shown are all individual scores for each pose as scattered points, as well as boxplots, where the box resembles the central quartiles including the median line, and the whiskers show minimum and maximum, respectively). n=49, see Source Data.

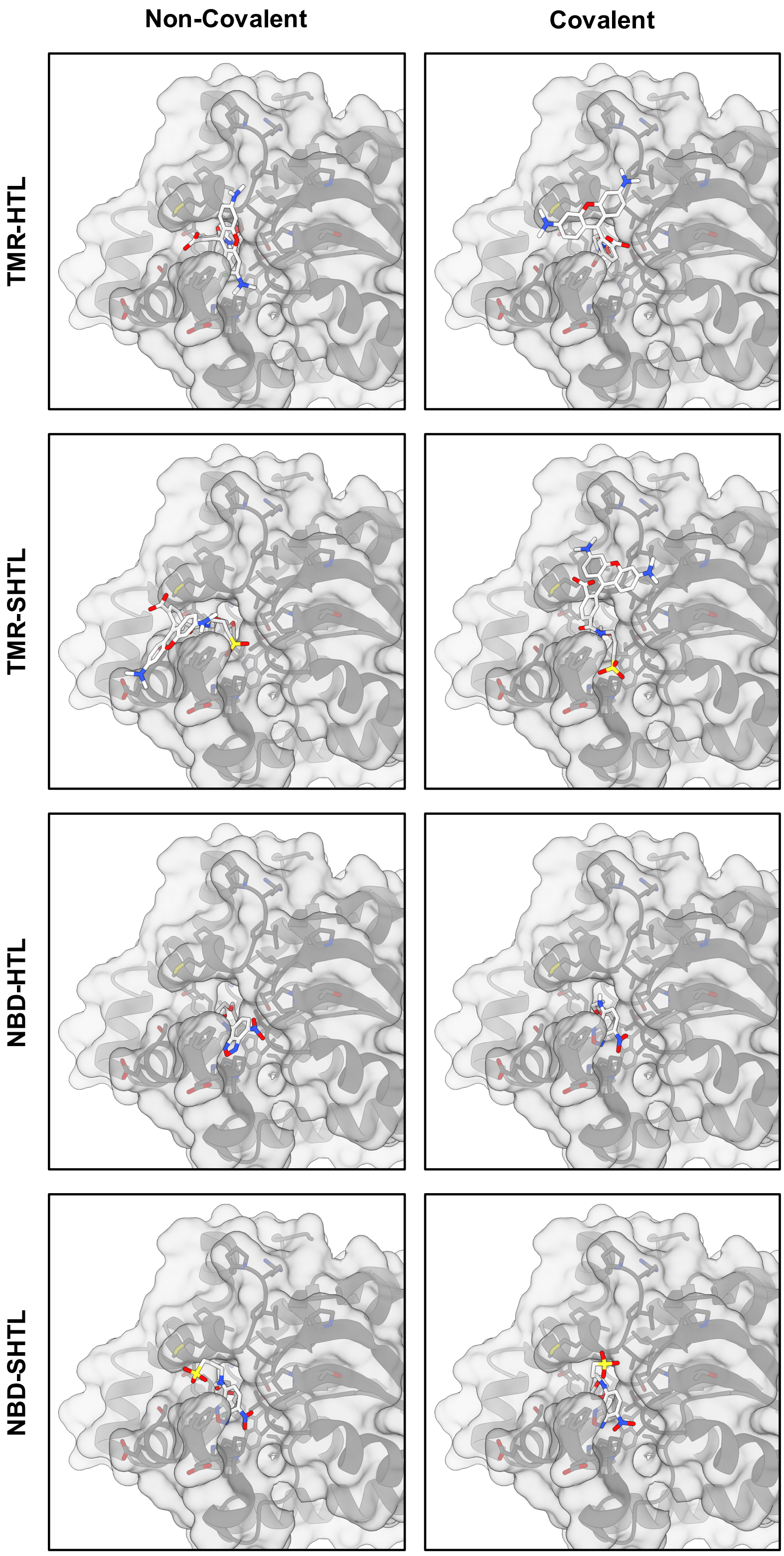

Supplementary Figure 4: Best Docking Conformations. For each ensemble, the CNN scores were used to rank all obtained poses and select the best geometry as the one having the highest affinity score. Each depiction shows the receptor (PDB-5UY1, HaloTag) as semi-transparent cartoon, as well as the ligand conformation (cyan). For each model, the residues within a 4.5 Å radius around the ligand are shown as wireframes. In the covalent poses, the conjugated residue ASP106 is rendered in cyan as well.

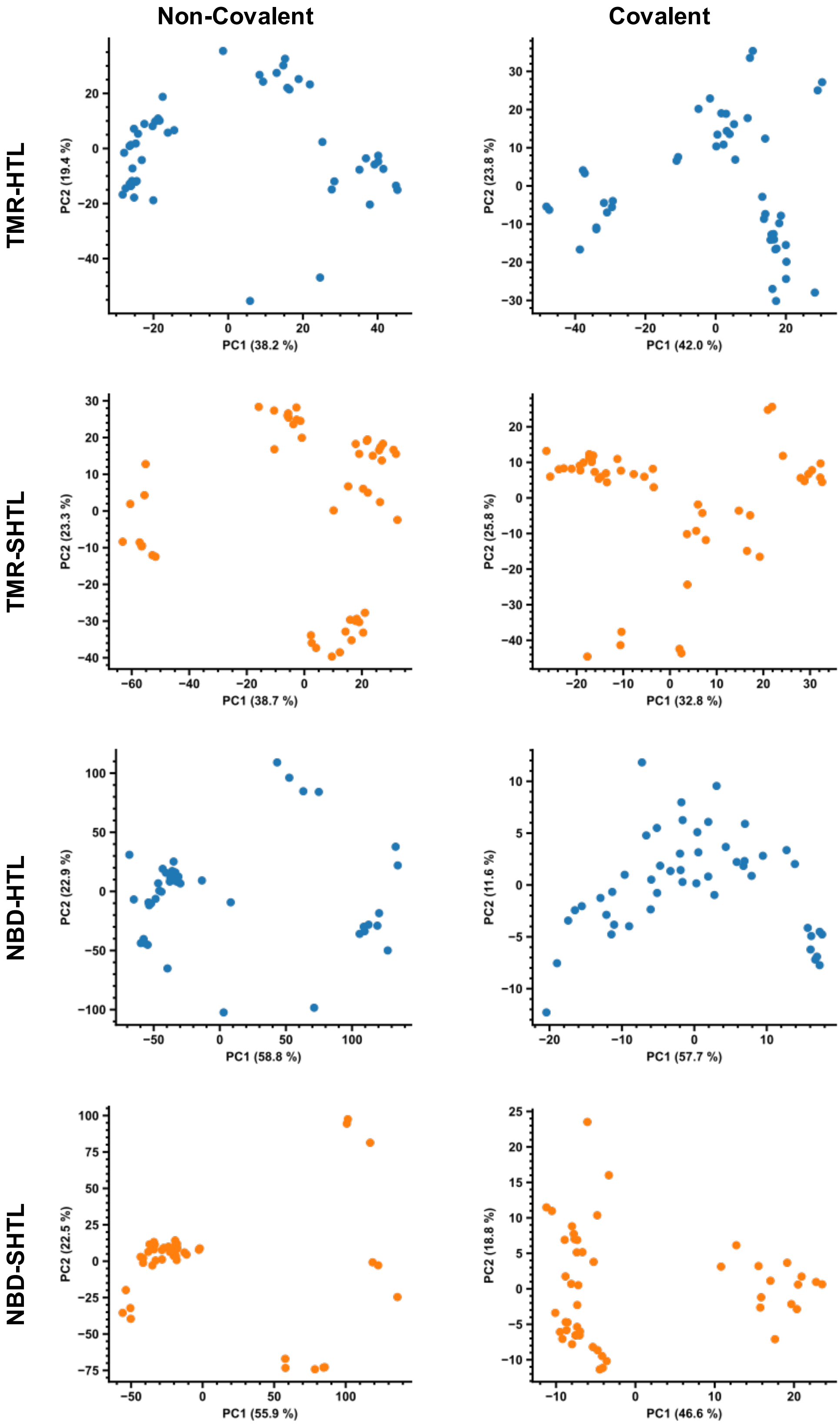

Supplementary Figure 5: Principal components for all ensembles. For each ensemble, all obtained poses were subjected to principal component analysis, whereas the cartesian coordinates of each ligand geometry were used as descriptors. Each pose is depicted as a point in the plane of the first two principal components, as selected by their respective explained variance (given on each axis in percent).

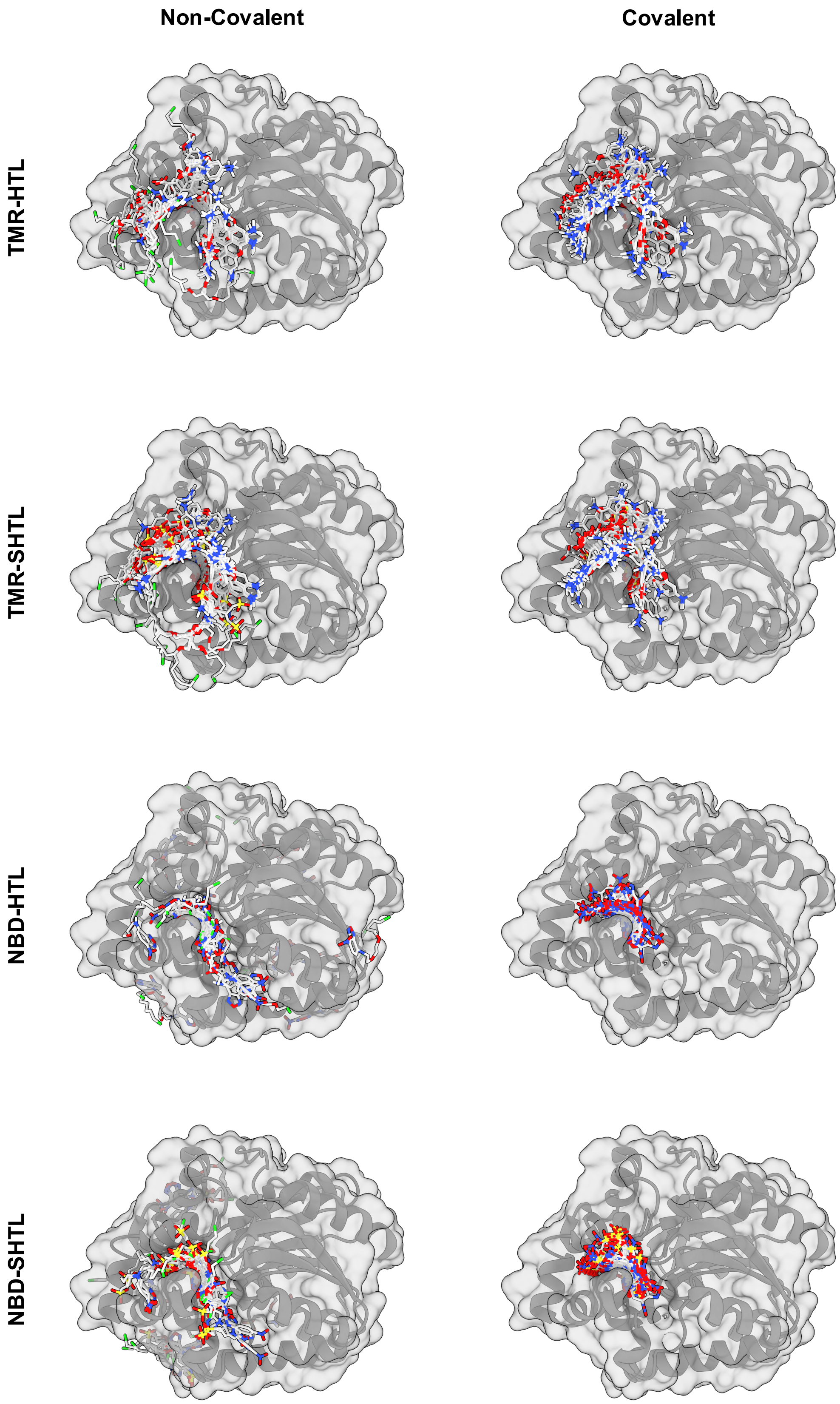

Supplementary Figure 6: Conformer Ensembles after Docking. For each ligand, the non-covalently and covalently docked conformer ensembles are depicted. The receptor (PDB-5UY1, HaloTag) is rendered as semi-transparent cartoon, whereas the ligand (including the conjugated ASP106) is rendered as stick models in cyan.

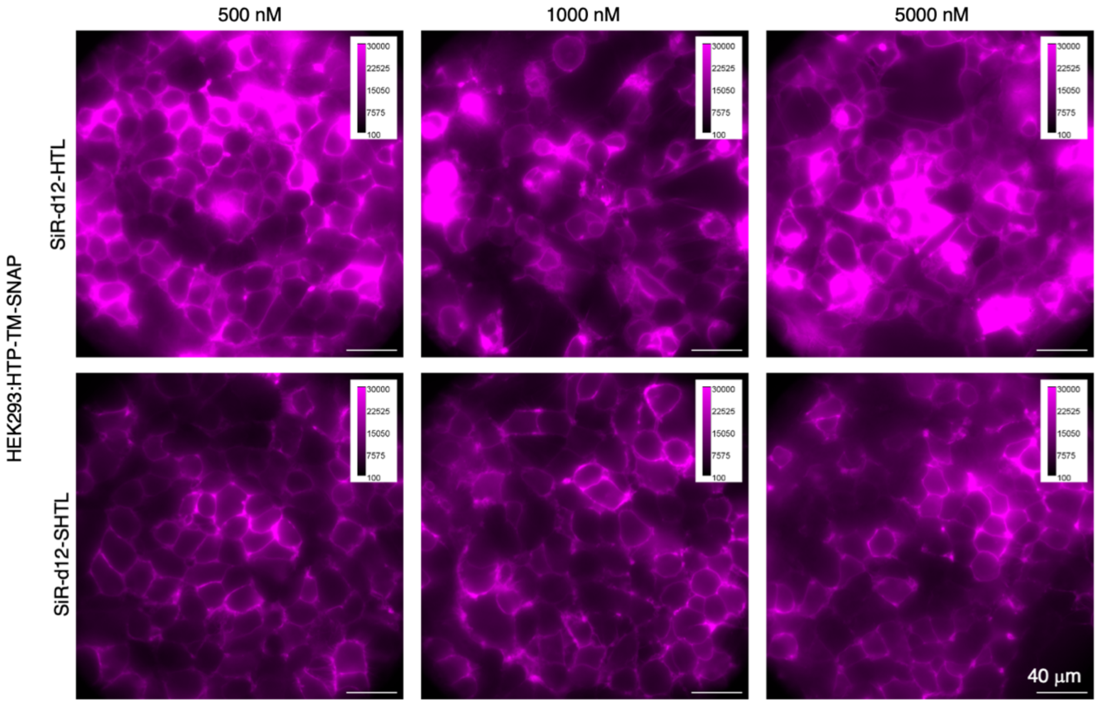

Supplementary Figure 7: Titration experiment using SiR-d12-HTL and SiR-d12-SHTL on live HTP-TM-SNAP transfected HEK293T cells. Imaging was performed for all conditions in N=3. For all images, scale bar = 40 mm.

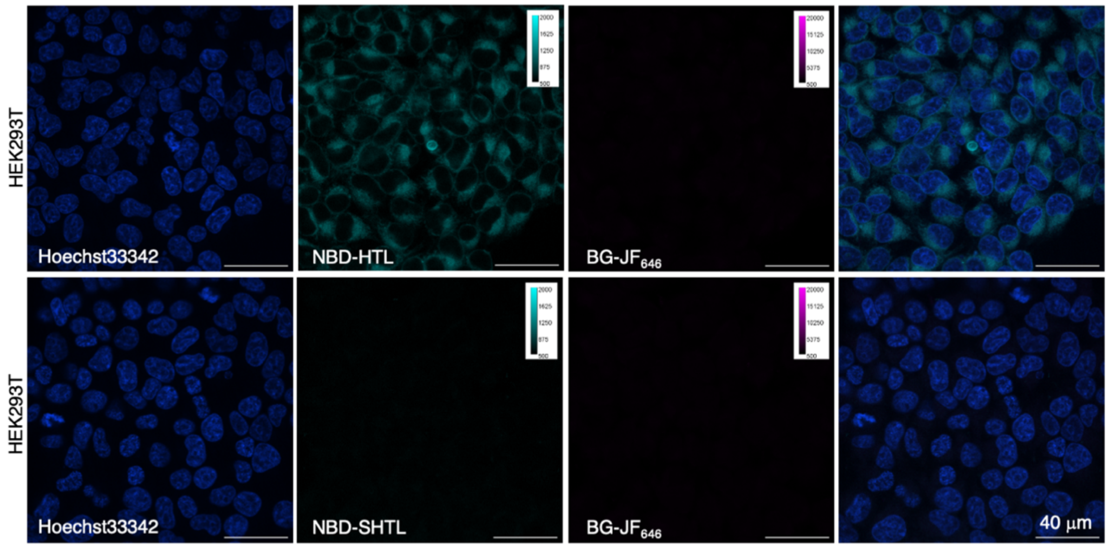

Supplementary Figure 8: Control experiments for confocal imaging using NBD-(S)HTL in non-transfected HEK293T cells. Imaging was performed for all conditions in N=3. For all images, scale bar = 40 mm.

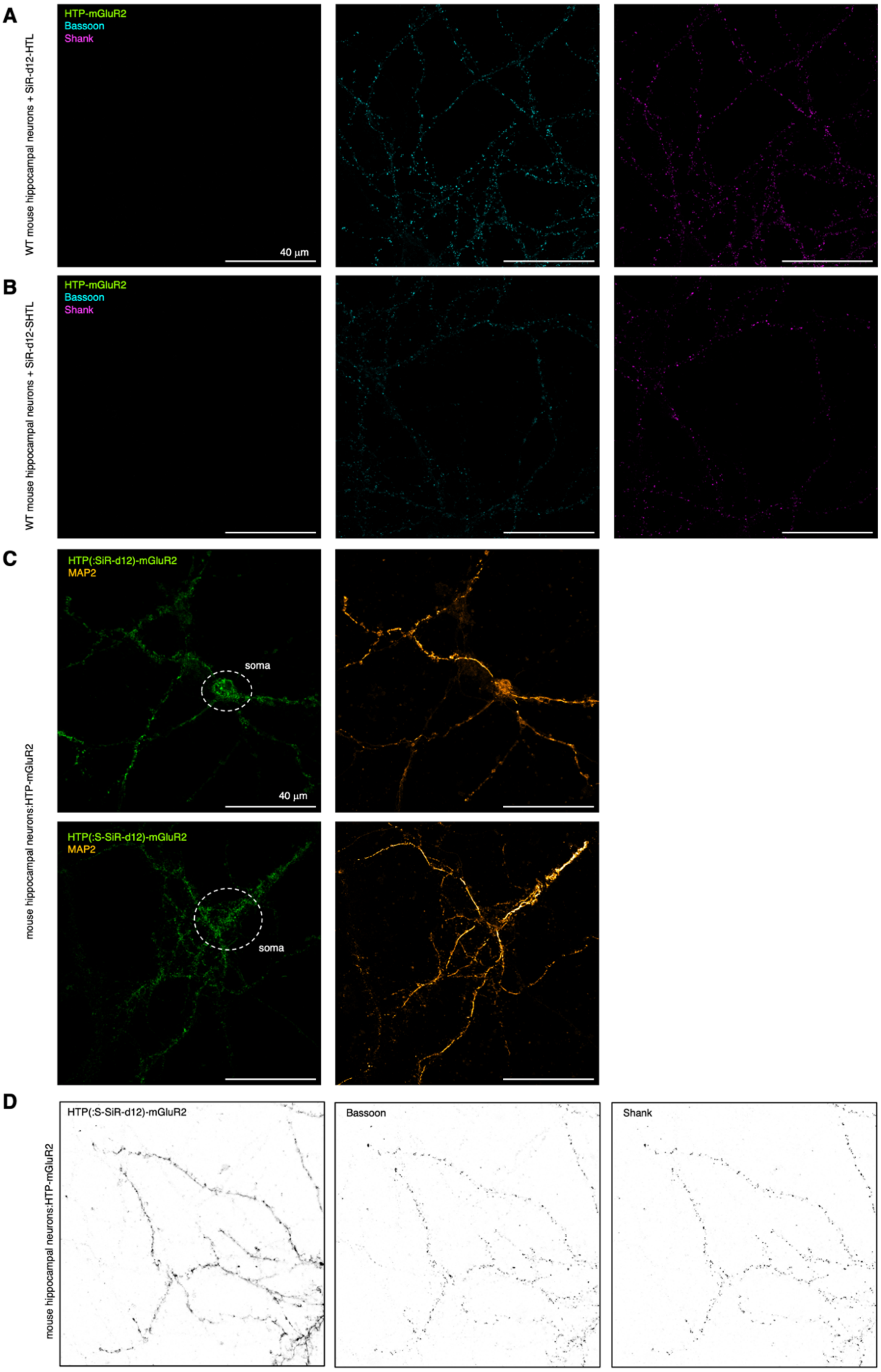

Supplementary Figure 9: Non-transduced neurons stained with SiR-d12-HTL **(A)** and SiR-d12-SHTL **(B). C)** MAP2 and HTP-mGluR2 single channel images from Figure 3D. **C)** Confocal imaging of HTP-mGluR2 transduced mouse hippocampal neurons cells with SiR-d12-SHTL (500 nM), and the pre- and postsynaptic markers Bassoon and Shank (scale bar = 40 µm). Imaging was performed for all conditions in N=3 preparations.

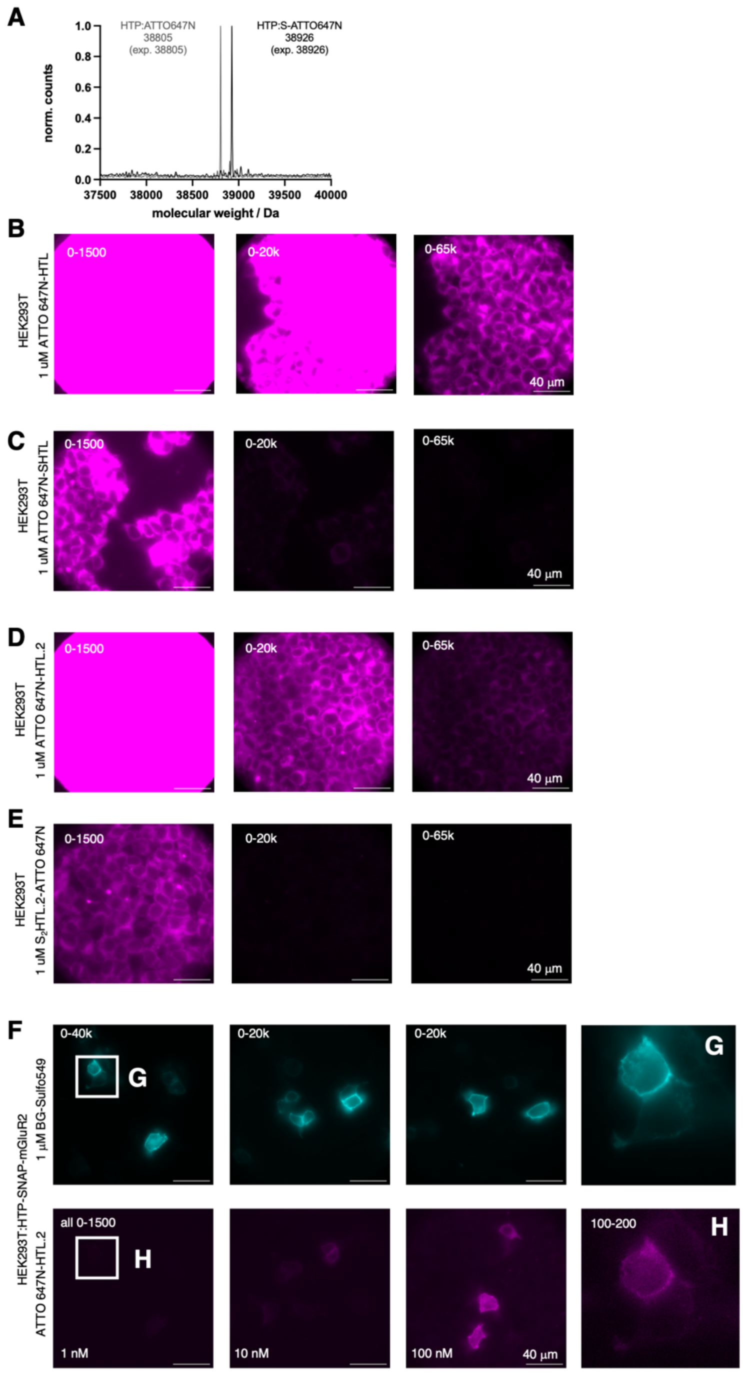

Supplementary Figure 10: A) Full protein mass spectrometry shows HTP-labelling with ATTO 647N-HTL and ATTO 647N-S_2HTL.2. **B-E)** HEK293T cells treated with 1 μM ATTO 647N-HTL (B), SHTL (C), HTL.2 (D) and S_2HTL.2 (E) with different brightness and contrast settings for comparison. **F)** SNAP-HTP-mGluR2 transfected HEK293T cells treated with BG-Sulfo549 (1 μM) and titrated with ATTO 647N-HTL. **G)** Zoom in of cells treated with BG-Sulfo549. **H)** As for (G) but with ATTO 647N-S_2HTL.2. Imaging was performed for all conditions in N=3 preparations. For all images, scale bar = 40 μm.

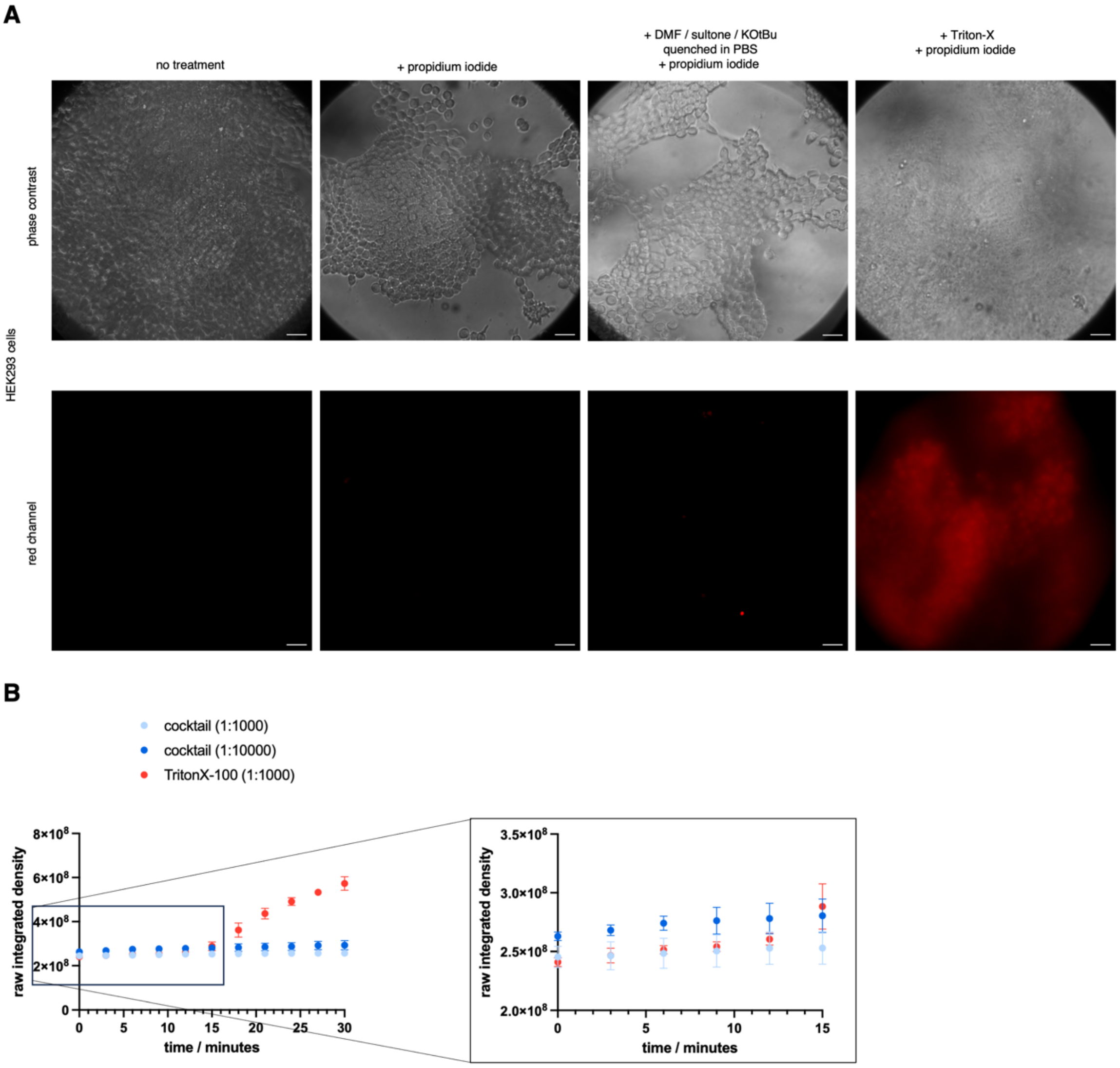

Supplementary Figure 11: PI assay using the *in situ* cocktail with propidium iodide. A) Widefield imaging of HEK293T cells after 5 minutes incubated with propidium iodide (1:250 of a 9 wt% solution), and additional cocktail or TritonX-100 as positive control. Scale bar = 40 micrometer. **B)** Integrated density of propidium iodide (1:1000 of a 9 wt% solution) over time shows cell death after 15 minutes, while different dilutions of the labelling cocktail does not lead to increased signal intensity. n=3 images. Mean±SD, see Source Data.

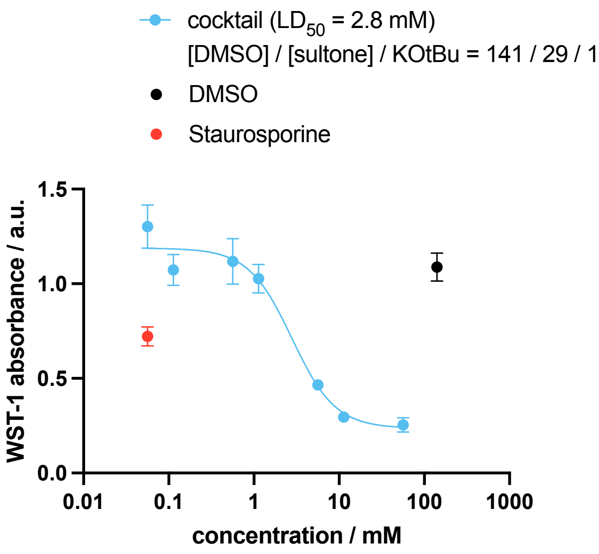

Supplementary Figure 12: WST-1 assay. HEK293T cells were seeded (40,000 cells/well) in a clear 96 well-plate and allowed to grow for 1 days in 100 uL DMEM supplemented with 10% FBS at 37 °C and 5% CO_2. Solutions were prepared in full medium, cells were aspirated before addition of the solutions. Incubation occurred over night. WST-1 (#MK400, Takara Bio) was added on top according to the manufacturer's instructions. Incubation was performed for another 2 hours before absorbance was read on a TECAN INFINITE M PLEX plate reader (λ_{Abs} = 440 nm) and corrected by subtraction ($\lambda_{Abs\ correct}$ = 660 nm). Plotting was performed in GraphPad Prism 8. Staurosporine and DMSO: n=3; Cocktails: n=6. See Source Data. Mean±SD. LD_{50} was calculated in Graphpad Prism 10.

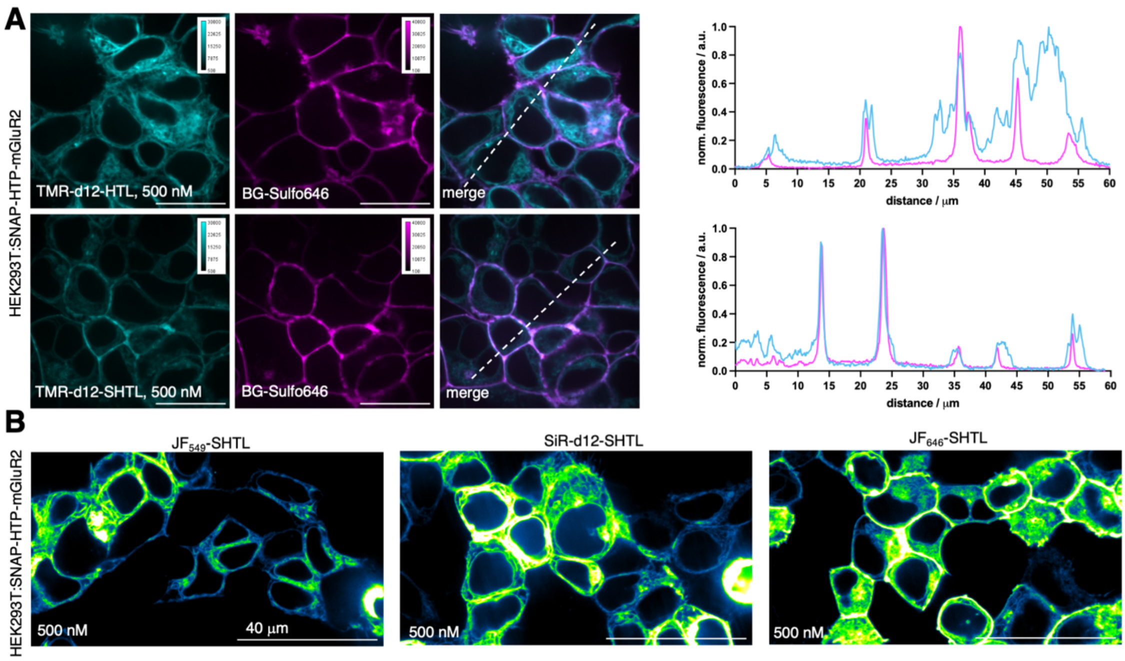

Supplementary Figure 13: Staining as for Figure 7, but with 1:1,000-fold dilution of the reaction mixture shows less clear surface staining. Imaging was performed for all conditions in N=3 preparations with n=3 images per condition. scale bar = 20 µm (A) and 40 µm (B).

5 Supplementary Tables

Supplementary Table 1. Multivariate regression results for correlation analysis of affinity scores vs. principal conformational variations, as obtained for all modelled ensembles of TMR-HTL and TMR-SHTL.

Docking Mode	Scoring	Ligand	*R* (affinity vs. PC1+PC2)	*p*-value
Non-Covalent	Empirical	TMR-HTL	0.282	4.72×10^{-2}
		TMR-SHTL	0.428	1.94×10^{-3}
	CNN	TMR-HTL	0.458	8.24×10^{-4}
		TMR-SHTL	0.918	6.93×10^{-21}
Covalent	Empirical	TMR-HTL	0.090	0.546
		TMR-SHTL	0.195	0.176
	CNN	TMR-HTL	0.288	4.99×10^{-2}
		TMR-SHTL	0.291	4.01×10^{-2}

Supplementary Table 2. Mann-Whitney U test results for paired comparisons between both ligands for all modelled ensembles of TMR-HTL and TMR-SHTL.

Docking Mode	Scoring	Mann-Whitney U	*p*-value
Non-Covalent	Empirical	1375	3.91×10^{-1}
	CNN	790	1.54×10^{-3}
Covalent	Empirical	1981	6.08×10^{-9}
	CNN	114	1.93×10^{-14}

Supplementary Table 3. Multivariate regression results for correlation analysis of affinity scores vs. principal conformational variations, as obtained for all modelled ensembles of NBD-HTL and NBD-SHTL.

Docking Mode	Scoring	Ligand	*R* (affinity vs. PC1+PC2)	*p*-value
Non-Covalent	Empirical	NBD-HTL	0.249	8.45×10^{-2}
		NBD-SHTL	0.185	1.98×10^{-1}
	CNN	NBD-HTL	0.299	3.71×10^{-2}
		NBD-SHTL	0.450	1.03×10^{-3}
Covalent	Empirical	NBD-HTL	0.656	7.16×10^{-3}
		NBD-SHTL	0.379	3.19×10^{-7}
	CNN	NBD-HTL	0.208	1.52×10^{-1}
		NBD-SHTL	0.366	9.60×10^{-3}

Supplementary Table 4. Mann-Whitney U test results for paired comparisons between both ligands for all modelled ensembles of NBD-HTL and NBD-SHTL.

**Docking Mode**	**Scoring**	**Mann-Whitney U**	***p*-value**
Non-Covalent	Empirical	1867	7.14×10^{-6}
	CNN	455	7.23×10^{-8}
Covalent	Empirical	2179	3.68×10^{-12}
	CNN	80	1.75×10^{-15}

6 One-step reaction protocol

Please cite Roßmann *et al.*, *Nat. Commun.* **2026**.

We recommend to work in a fume hood.

- Weigh in 1.0 mg KO*t*Bu (Aldrich: #659878) into an Eppendorf tube.
- Add 104 uL of DMSO (we recommend single use ampules: e.g. Carl Roth: #AE56.3, 10 x 0.75 mL) to make a 100 mM stock solution.

- Add 1,3-propane sultone into an Eppendorf tube and warm on a shaker to 35 °C to obtain a liquid (mp = 32 °C)
- Add 4 uL of DMSO solution to 5 nmol of the dye-HTL conjugate (e.g. Promega: #HT1020 for JF_{549}-HTL and #HT1060 for JF_{646}-HTL – in this case the 5 x 1 nmol aliquots can be pooled), briefly vortex to ensure full dissolution and spin down.
- Add 1 uL of liquid sultone into the DMSO solution and pipette up and down to ensure full mixing.
- Allow incubation for 5 minutes at room temperature.

- Add 5 uL PBS to the reaction mixture and briefly vortex and spin down.
- The reaction is completed and quenched. Dilute 1:10,000 in medium of choice, aspirate cells medium and replace with labelling solution for 30 minutes at 37 °C. Wash cells once and proceed with protocol of choice (e.g., fixation, imaging).

<u>Concentrations during the steps:</u>

[DMSO] = 14.1 M

[1,3-propane sultone] = 10.8 M

[KO*t*Bu] in DMSO = 100 mM

Adding sultone to DMSO:

[DMSO] = 11.3 M

[1,3-propane sultone] = 2.16 M

[KO*t*Bu] = 80 mM

Quench with PBS, hydrolyzing sultone to sulfonic acid:

[DMSO] = 5.64 M

[3-hydroxypropanesulfonic acid] = 1.08 M

[KO*t*Bu] = 40 mM

Dilute 1:10000:

[DMSO] = 564 μM

[3-hydroxypropanesulfonic acid] = 108 μM

[KO*t*Bu] = 4 μM

7 References

[1] D. Weininger, "SMILES, a chemical language and information system. 1. Introduction to methodology and encoding rules" *J. Chem. Inf. Comput. Sci.* **1988**, *28*, 31–36.

[2] D. Weininger, A. Weininger, J. L. Weininger, "SMILES. 2. Algorithm for generation of unique SMILES notation" *J. Chem. Inf. Comput. Sci.* **1989**, *29*, 97–101.

[3] D. Weininger, "SMILES. 3. DEPICT. Graphical depiction of chemical structures" *J. Chem. Inf. Comput. Sci.* **1990**, *30*, 237–243.

[4] N. M. O'Boyle, M. Banck, C. A. James, C. Morley, T. Vandermeersch, G. R. Hutchison, "Open Babel: An open chemical toolbox" *Journal of Cheminformatics* **2011**, *3*, 33.

[5] T. A. Halgren, "Merck molecular force field. I. Basis, form, scope, parameterization, and performance of MMFF94" *Journal of Computational Chemistry* **1996**, *17*, 490–519.

[6] F. Neese, "Software Update: The ORCA Program System—Version 6.0" *WIREs Computational Molecular Science* **2025**, *15*, e70019.

[7] F. Neese, "An improvement of the resolution of the identity approximation for the formation of the Coulomb matrix" *J Comput Chem* **2003**, *24*, 1740–1747.

[8] F. Neese, F. Wennmohs, A. Hansen, U. Becker, "Efficient, approximate and parallel Hartree–Fock and hybrid DFT calculations. A 'chain-of-spheres' algorithm for the Hartree–Fock exchange" *Chemical Physics* **2009**, *356*, 98–109.

[9] Y. Liu, K. Miao, N. P. Dunham, H. Liu, M. Fares, A. K. Boal, X. Li, X. Zhang, "The Cation−π Interaction Enables a Halo-Tag Fluorogenic Probe for Fast No-Wash Live Cell Imaging and Gel-Free Protein Quantification" *Biochemistry* **2017**, *56*, 1585–1595.

[10] E. C. Meng, T. D. Goddard, E. F. Pettersen, G. S. Couch, Z. J. Pearson, J. H. Morris, T. E. Ferrin, "UCSF ChimeraX: Tools for structure building and analysis" *Protein Science* **2023**, *32*, e4792.

[11] M. V. Shapovalov, R. L. Dunbrack, "A smoothed backbone-dependent rotamer library for proteins derived from adaptive kernel density estimates and regressions" *Structure* **2011**, *19*, 844–858.

[12] A. T. McNutt, P. Francoeur, R. Aggarwal, T. Masuda, R. Meli, M. Ragoza, J. Sunseri, D. R. Koes, "GNINA 1.0: molecular docking with deep learning" *J Cheminform* **2021**, *13*, 43.

[13] J. Sunseri, D. R. Koes, "Virtual Screening with Gnina 1.0" *Molecules* **2021**, *26*, 7369.
