# Supplementary material for: A general one-step protocol to generate impermeable fluorescent HaloTag substrates for in situ live cell application and super-resolution imaging
Source: Nat Commun. 2026 Jan 12;17:426. doi: 10.1038/s41467-025-68134-0 (PMC12796469; doi:10.1038/s41467-025-68134-0)
Supplement: Supplementary file 2 — Reporting Summary [file 41467_2025_68134_MOESM2_ESM.pdf]

nature portfolio

Corresponding author(s): Johannes Broichhagen

Last updated by author(s): Apr 1, 2025

Reporting Summary

Nature Portfolio wishes to improve the reproducibility of the work that we publish. This form provides structure for consistency and transparency in reporting. For further information on Nature Portfolio policies, see our Editorial Policies and the Editorial Policy Checklist.

Statistics

For all statistical analyses, confirm that the following items are present in the figure legend, table legend, main text, or Methods section.

n/a	Confirmed	
☐	☒	The exact sample size (*n*) for each experimental group/condition, given as a discrete number and unit of measurement
☐	☒	A statement on whether measurements were taken from distinct samples or whether the same sample was measured repeatedly
☐	☒	The statistical test(s) used AND whether they are one- or two-sided<br>*Only common tests should be described solely by name; describe more complex techniques in the Methods section.*
☒	☐	A description of all covariates tested
☒	☐	A description of any assumptions or corrections, such as tests of normality and adjustment for multiple comparisons
☐	☒	A full description of the statistical parameters including central tendency (e.g. means) or other basic estimates (e.g. regression coefficient) AND variation (e.g. standard deviation) or associated estimates of uncertainty (e.g. confidence intervals)
☐	☒	For null hypothesis testing, the test statistic (e.g. F, t, r) with confidence intervals, effect sizes, degrees of freedom and P value noted<br>*Give P values as exact values whenever suitable.*
☒	☐	For Bayesian analysis, information on the choice of priors and Markov chain Monte Carlo settings
☒	☐	For hierarchical and complex designs, identification of the appropriate level for tests and full reporting of outcomes
☒	☐	Estimates of effect sizes (e.g. Cohen's *d*, Pearson's *r*), indicating how they were calculated

Our web collection on statistics for biologists contains articles on many of the points above.

Software and code

Policy information about availability of computer code

Data collection	- Confocal and STED images were acquired with a Leica SP8 STED microscope operated by Leica Application Suite X v3.5.10.29396 from Leica Microsystems.<br>- Widefield images were acquired on a Nikon Ti Eclipse with perfect focus system automated stage operated by operated by NIS Elements v5.4.42 .<br>- Confocal images were acquired by a Nikon CSU-X1 microscope operated by NIS Elements v5.4.42<br>- Quantum Yields were recorded with a Hamamatsu Quantaurus operated the PLQY Software (HPK, U6039-05 Version 4.6.0).<br>- Fluorescent spectra were acquired on a Tecan Infinite 2000 Pro from Tecan Trading AG.<br>- Mass spectra were acquired on a Thermo Fisher Scientific Orbitrap Fusion mass spectrometer.<br>- Modelling: ORCA v6.1.0; OpenBabel v3.1.0; SciPy v1.13.1; NumPy v1.26.4; Matplotlib v3.9.0; ChimeraX v1.10.1; GNINA v1.1
Data analysis	The following software was used for data analysis:<br>- Microsoft Excel (version 16.78.3) for data storage and processing<br>- GraphPad Prism 10 for statistical analysis and plotting<br>- FIJI/ImageJ2 (version 2.14.0/1.54p) for image analysis<br>No custom code was used.

For manuscripts utilizing custom algorithms or software that are central to the research but not yet described in published literature, software must be made available to editors and reviewers. We strongly encourage code deposition in a community repository (e.g. GitHub). See the Nature Portfolio guidelines for submitting code & software for further information.

Data

Policy information about availability of data

All manuscripts must include a data availability statement. This statement should provide the following information, where applicable:

- Accession codes, unique identifiers, or web links for publicly available datasets
- A description of any restrictions on data availability
- For clinical datasets or third party data, please ensure that the statement adheres to our policy

The source data has been uploaded with the submission.

Research involving human participants, their data, or biological material

Policy information about studies with human participants or human data. See also policy information about sex, gender (identity/presentation), and sexual orientation and race, ethnicity and racism.

Reporting on sex and gender	n/a
Reporting on race, ethnicity, or other socially relevant groupings	n/a
Population characteristics	n/a
Recruitment	n/a
Ethics oversight	n/a

Note that full information on the approval of the study protocol must also be provided in the manuscript.

Field-specific reporting

Please select the one below that is the best fit for your research. If you are not sure, read the appropriate sections before making your selection.

[x] Life sciences [ ] Behavioural & social sciences [ ] Ecological, evolutionary & environmental sciences

For a reference copy of the document with all sections, see nature.com/documents/nr-reporting-summary-flat.pdf

Life sciences study design

All studies must disclose on these points even when the disclosure is negative.

Sample size	All sample sizes were chosen based on estimated effect sizes determined from from preliminary experiments and published literature. Statistical analysis were used to verify their sufficiency. Generally, all sample sizes were at least n=3 for each condition, with representative data shown in the manuscript.
Data exclusions	No samples were excluded from analysis.
Replication	All experiments were repeated independently multiple times to ensure reproducibility, and all key experiments were analyzed for statistical significance.
Randomization	Randomization was not relevant to this study, because all cells used throughout the study had to be differentially treated and analyzed in parallel to minimize experimental variation. Hence, their identity was known to the investigator.
Blinding	not appliable.

Reporting for specific materials, systems and methods

We require information from authors about some types of materials, experimental systems and methods used in many studies. Here, indicate whether each material, system or method listed is relevant to your study. If you are not sure if a list item applies to your research, read the appropriate section before selecting a response.

Materials & experimental systems

n/a	Involved in the study	
	x	Antibodies
	x	Eukaryotic cell lines
x		Palaeontology and archaeology
	x	Animals and other organisms
x		Clinical data
x		Dual use research of concern
x		Plants

Methods

n/a	Involved in the study	
x		ChIP-seq
x		Flow cytometry
x		MRI-based neuroimaging

Antibodies

Antibodies used

Antibody number. Antibody; Source; Dilution used; RRID; Identifier; clone ID (when relevant)

Ab 1. Guinea pig polyclonal Shank 2; Synaptic Systems; 1:250; AB_2619861; 162 204; n/a
Ab 2. Chicken polyclonal MAP2; Novus Biologicals; 1:1000; AB_2138178; NB300-213; n/a
Ab 3. Mouse monoclonal anti Bassoon; Abcam; 1:250; AB_3182580; SAP7F407; SAP7F407

Secondary Abs for immunostaining
Ab 4. Donkey anti-guinea Pig IgG (H+L) CF488; Biotium; 1:500; AB_10853117; 20169-1
Ab 5. Goat anti-chicken Alexa 405; Abcam 1:500; AB_2890171; Ab175674
Ab 6. Donkey anti-mouse IgG (H+L) Alexa594; Invitrogen; 1:500; AB_2762826; A32744

Validation

Ab 1. Guinea pig polyclonal Shank 2; Synaptic Systems; 1:250; AB_2619861; 162 204 Manufacturer website: Antibody was validated using KO samples (PMID: 2997098)

Ab 2. Chicken polyclonal MAP2; Novus Biologicals; 1:1000; AB_2138178; NB300-213
Manufacturer website: Knockdown Validated (PMID: 32294442). More publications in https://www.novusbio.com/products/map2antibody_nb300213?srsltid=AfmBOorEtC2sqRWpEaMtjX36c2y8L3tkHsKuvStTIAsTiKx6DkunwKBB#reviews-publications

Ab 3. Mouse monoclonal anti Bassoon; Abcam; 1:250; AB_3182580; SAP7F407; SAP7F407
Validated against a partial gene deletion mutant in PMID: 21092861, supplemental information section.

Ab 4. Donkey anti-guinea Pig IgG (H+L) CF488; Biotium; 1:500; AB_10853117; 20169-1
Manufacturer website: Cross adsorption against Bovine, Chicken, Goat, Horse, Human, Mouse, Rabbit, Rat, Sheep, Syrian hamster

Ab 5. Goat anti-chicken Alexa 405; Abcam 1:500; AB_2890171; Ab175674
Manufacturer website: This antibody was isolated by affinity chromatography using antigen coupled to agarose beads.

Ab 6. Donkey anti-mouse IgG (H+L) Alexa594; Invitrogen; 1:500; A32744
Manufacturer website: 'Cross Adsorption Against bovine, chicken, goat, guinea pig, hamster, horse, human, rabbit, rat, and sheep serum'

Eukaryotic cell lines

Policy information about cell lines and Sex and Gender in Research

Cell line source(s): HEK293 cells were obtained from German Collection of Microorganism and Cell Cultures

Authentication: Cell lines from ATCC are regularly authenticated by STR profiling and were used by us without further authentication.

Mycoplasma contamination: Cell lines were not contaminated and were regularly tested for mycoplasma contamination.

Commonly misidentified lines (See ICLAC register): No commonly misidentified lines were used.

Animals and other research organisms

Policy information about studies involving animals; ARRIVE guidelines recommended for reporting animal research, and Sex and Gender in Research

Laboratory animals: Mus musculus: C57BL/J6 wild type. Mice were housed under standardized conditions (12 h light/dark cycle; 22-24 °C; 55±15% humidity ad libitum access to standard diet and water).

Wild animals	n/a
Reporting on sex	The sex of the animals was not considereed in the study design and a sex- and gender-based analysis was not performed in this study.
Field-collected samples	n/a
Ethics oversight	All the experiments performed in Berlin complied with European law and the state of Berlin animal welfare body (LAGeSo).

Note that full information on the approval of the study protocol must also be provided in the manuscript.

Plants

Seed stocks	n/a
Novel plant genotypes	n/a
Authentication	n/a
